# Angular-Dependent Energy-Saving Smart Windows

**DOI:** 10.1021/acsnano.5c13103

**Published:** 2025-09-15

**Authors:** Keunhyuk Ryu, Guanya Wang, Vijay Shankar Sridharan, Shancheng Wang, ZhiLi Dong, Shuang Zhang, Yi Long

**Affiliations:** † School of Materials Science and Engineering, 54761 Nanyang Technological University, 639798 Singapore, Singapore; ‡ Department of Electronic Engineering, 26451 The Chinese University of Hong Kong, 999077 New Territories, Hong Kong SAR, China; § New Cornerstone Science Laboratory, Department of Physics, University of Hong Kong, 999077 Pokfulam, Hong Kong, China

**Keywords:** phase change material, seasonal solar variation, dual-band optical modulation, passive thermal regulation, metasurface, smart windows

## Abstract

Windows are responsible for nearly 50% of the building’s heat loss. Most current smart window designs solely consider the season-accompanied temperature change but often overlook the solar zenith angle variation. This work addresses this critical gap by leveraging the potential of dynamic metasurfaces and engineering the angular and thermal dual-responsiveness into structural engineering via scalable and industrially compatible mesh printing and spray-coating. The season-dependent solar/thermal radiation dual-modulation smart window, which is composed of a structured reconfigured vanadium dioxide (VO_2_) array-based Fabry–Perot resonator, dynamically responds to variations in both solar zenith angle and temperature. The proposed smart window achieves promising luminance transmittance (36.8%), solar modulation (30.8%), and broadband infrared emissivity modulation (0.4). It outperforms the commercial low-emissivity glass and the state-of-the-art designs in energy-saving performance simulation and daylight illumination. Furthermore, the device shows promising color rendering performance and near-daylight color temperature, ensuring superior visual comfort and color neutrality over conventional smart windows. The integration of metasurfaces and phase-change materials provides a promising strategy to dynamically modulate optical responses across different wavelengths, which could have potentially wide applications not limited to energy-saving building facades.

## Graphical abstract

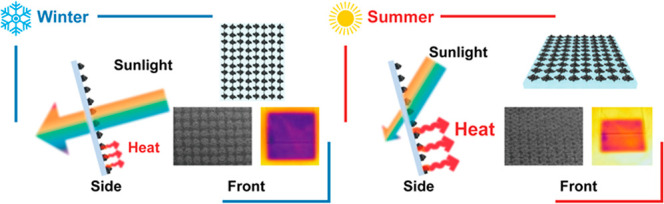

## Introduction

A metasurface is a two-dimensional array of subwavelength structures designed to manipulate electromagnetic waves, which can control light in ways that traditional optics cannot.^1–5^ Most traditional metasurfaces are made from static materials, which constrain their potential, especially the dynamic optical modulation needed in energy conservation applications.^6–12^ On the other hand, the International Energy Agency predicts that energy demand in the construction sector could rise by 50% by 2050 if no energy efficiency improvements are made.^13,14^ Notably, windows are considered as the least energy efficient part of buildings, attributing 47% of the building’s heat loss.^15–21^ Driven by the demand for building energy conservation, research on smart windows that modulate heat management in response to stimuli has attracted significant interest.^22^ Smart windows can be categorized according to the stimuli they respond to, such as thermo-, electro-, photo-, and mechano-responsive smart windows.^23^ Among smart windows, thermochromic smart windows are particularly competitive due to their advantages, such as simple structure, independence from extra energy sources, and rational stimulus choice. The performance indexes are luminous transmittance (*T*_lum_, 360–780 nm), solar modulation ability (Δ*T*_sol_, 360–2500 nm), near-infrared modulation ability (Δ*T*_NIR_, 790–2500 nm), and more recently, a broadband infrared (broadband IR) emissivity modulation ability (Δε_broadband_, 2.5–20 μm).^24^

However, most smart window designs focus primarily on responding to various stimuli while neglecting the variability in the seasonal solar zenith angles (θ). Specifically, most smart windows assume a static, perpendicular θ. In real applications, especially for regions with distinct seasons, the θ and solar radiation intensity change dynamically due to the Earth’s rotation and revolution around the sun (Scheme 1a). Meanwhile, the solar radiation intensity in midlatitude regions peaks near solar noon (10 a.m. to 2 p.m.) (Scheme 1b and Figure S1). For example, in Seoul, solar radiation during solar noon accounts for approximately 60% and 80% of the total daily solar radiation in June and December, respectively. A similar pattern of solar radiation intensity is also observed in other representative midlatitude cities (Figure S1), underscoring the critical importance of solar management during solar noon for effective building energy savings.^25,26^ Therefore, θ at solar noon was chosen as the representative condition for this study. In midlatitude regions, the θ at solar noon exhibits a seasonal variation of approximately 60° between summer and winter (Scheme 1c). To conceptually and comprehensively capture the seasonal variation in θ across midlatitude regions, representative values were set to 90° for winter and 30° for summer arbitrarily. Previously, a VO_2_-grating structure was designed to take season-dependent θ variation into consideration,^25^ which, however, could not regulate outgoing thermal radiation, limiting its energy-saving performance.

**Scheme 1:**
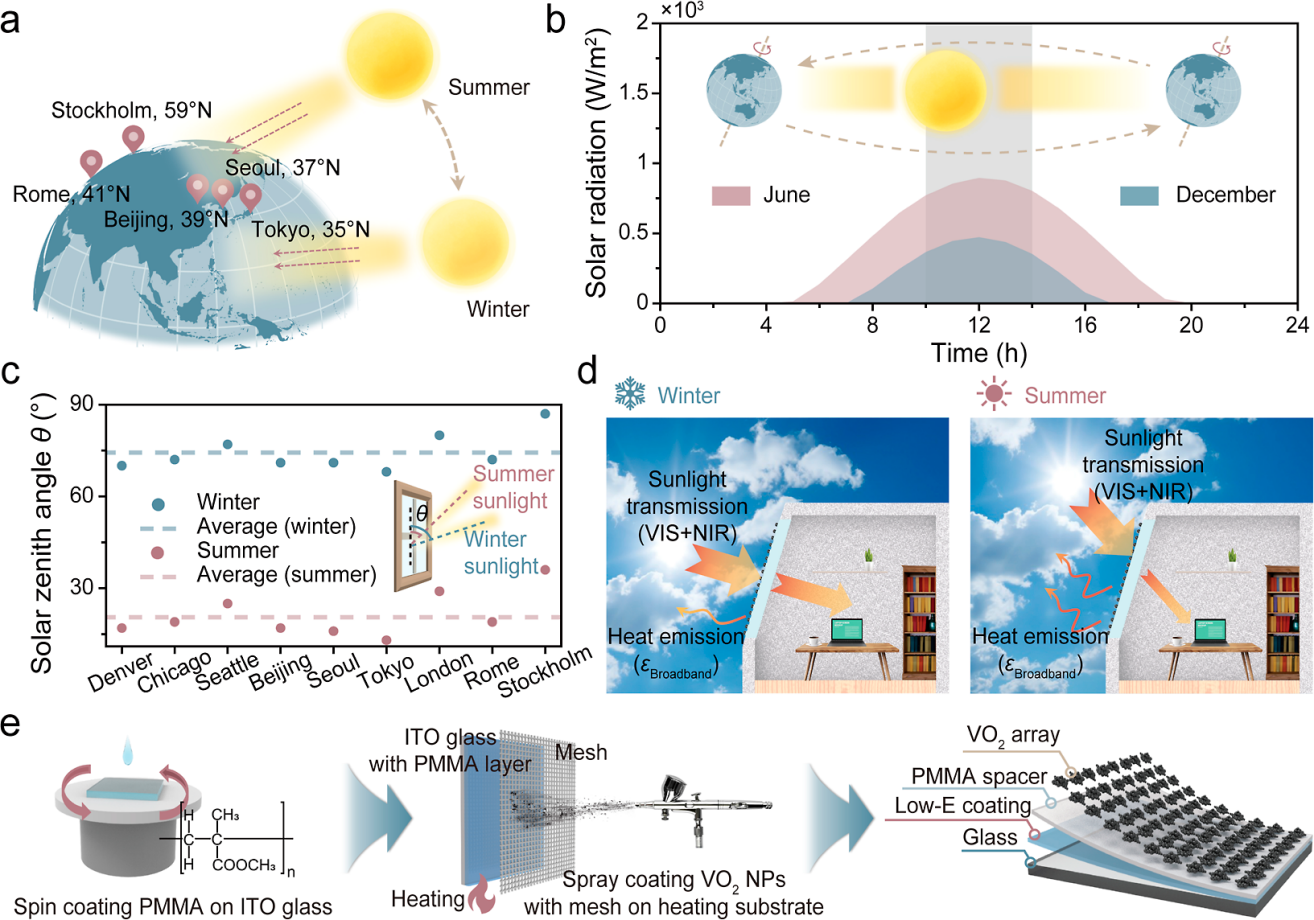
(a) Schematic Illustration of Seasonal Variations in θ between Summer and Winter in Midlatitude Regions of the Northern Hemisphere; (b) Representative Daily Solar Radiation Intensity Profile for Seoul, a Midlatitude City; Inset: Schematic Showing the Earth’s Orbital Revolution and Axial Tilt Responsible for Solar Angle Variations between June and December; Gray Shadow Indicates the Solar Noon Period (10 a.m. to 2 p.m.); (c) Variation of θ According to Seasonal Changes in Midlatitude Regions during Solar Noon; Inset: Definition of θ Relative to the Window Surface; (d) Operating Principle of Season-Dependent Solar/Thermal Radiation Dual-Modulation Smart Window in Winter and Summer; (e) Schematic Illustration of the Mesh Printing and Spray-Coating Process and the VO_2_ Array-Based F–P Resonator Structure

Thereby, we report a smart window design featuring a metasurface-reconfigured VO_2_ array-based Fabry–Perot resonator (F–P resonator) manufactured via an industrially compatible mesh printing and spray-coating strategy. The VO_2_ array and F–P resonator that is formed by stacking the VO_2_ array, solar and broadband IR transparent poly­(methyl methacrylate) (PMMA), and underneath low-emissivity (low-E) indium tin oxide (ITO) coating enable sunlight/outgoing thermal radiation dual-modulation across different seasons (Scheme 1d). With a tilted configuration of the smart window, conceptually designed to accommodate seasonal variations in θ, winter sunlight at larger angles could pass through the window to heat the room by a highly near-infrared (NIR, 780–2500 nm) transparent monoclinic VO_2_ (VO_2_(M)) array. In the meantime, the high broadband IR transparency of VO_2_(M) and PMMA leads to the exposure of the underneath ITO low-E layer, resulting in suppressed outgoing thermal radiation. In the summer, VO_2_ undergoes a phase transition from VO_2_(M) to NIR absorbance rutile VO_2_ (VO_2_(R)). Accompanied by an increased effective blocking volume due to the smaller θ, sunlight is blocked by VO_2_(R). Simultaneously, the F–P resonator prompts the outgoing thermal radiation by exhibiting high broadband IR emissivity (ε_broadband_). Beyond dual-wavelength modulation ability, the use of a VO_2_ array—rather than a continuous VO_2_ film—further allows for improved *T*_lum_ for daylight illumination. As the VO_2_ array-based season-dependent dual-modulation smart window is fabricated via the solution-based mesh printing and spray-coating method (Scheme 1e), it demonstrates a promising capability for large-scale production. The smart window shows competitive performance with a *T*_lum_ value of 36.8%, a Δ*T*_sol_ value of 30.8%, and a Δε_broadband_ value of 0.41. Moreover, in the actual-sized building energy-saving simulation in distinct season regions such as Seattle, London, and Seoul, the proposed smart window outperforms commercial low-E glass and the state-of-the-art.^24,25^ The periodic VO_2_ array-based smart window, engineered for seasonal variations in θ and temperature, not only optimizes energy efficiency but also offers strong potential for large-scale production through a facile spray process, offering a promising route toward dynamic thermal metasurface integration in architecture and beyond.

## Results and Discussion

### Season-Dependent Solar/Thermal Radiation Dual-Modulation Performance

Finite-difference time-domain (FDTD) simulation was conducted to understand the metasurface effects at different θ and temperatures. The VO_2_ array-based F–P resonator is expected to demonstrate sunlight/outgoing thermal radiation dual-modulation in response to variation in θ and temperature. By numerically simulating the winter (θ = 90°, low temperature (LT) = 30 °C) and summer (θ = 30°, high temperature (HT) = 90 °C) of the VO_2_ array-based F–P resonator, the results indicate the designed VO_2_ array-based F–P resonator structure shows high transmittance across the visible and NIR regions (31.4% and 57.7% for 500 and 1000 nm, respectively), while the emissivity is low (0.45) in the winter scenario. On the other hand, in the summer scenario, the device’s transmittance dramatically decreases to ∼25%, while its emissivity increases accordingly (Figure 1a). The propagation of light with different wavelengths in the structure mapped via FDTD electromagnetic field simulation is visualized in Figure 1b,c. In the winter scenario, a large fraction of the incident visible light (500 nm in the FDTD simulation) and NIR (1000 nm in the FDTD simulation) can be transmitted through the structure (Figure 1b­(i,ii)). In the summer scenario, due to the phase change of VO_2_ and increased effective blocking volume, the incident visible light and NIR are strongly attenuated by the structure (Figure 1b­(iii,iv)). As a result, the intensity of the light passing through the structure in summer is weaker than that in the winter. Meanwhile, in the winter scenario, incident radiation at 10 μm is largely reflected by the structure, as indicated by the strong electric field intensity represented by bright colors in the simulation (Figure 1c­(i)). This high field intensity corresponds to high broadband IR reflection, as the broadband IR transparent VO_2_(M) and PMMA layers expose the underlying highly reflective low-E surface. In contrast, under the summer scenario, the 10 μm radiation is strongly absorbed by the F–P resonator formed by VO_2_(R), PMMA, and ITO, leading to a reduced electric field intensity—shown as near-blue colors—which indicates strong absorption and, consequently, high thermal emissivity (Figure 1c­(ii)). These simulation results highlight seasonal-dependent optical responses of the VO_2_ array-based F–P resonator. Scanning electron microscopy (SEM) images of the VO_2_ arrays fabricated with different sizes of mesh are shown in Figure 1d. With the increase in mesh opening size from 57 to 209 μm, the size of the VO_2_ island increases correspondingly (Figure 1d­(i–iv)). The energy-dispersive X-ray spectroscopy (EDS) elemental maps show the elemental distribution in a periodic VO_2_ array on the substrate (Figures 1e and S3e); green, magenta, and yellow colors represent vanadium (V), silicon (Si), and oxygen (O), respectively. The EDS result confirms that a uniform periodic VO_2_ array is successfully formed with the mesh printing and spray-coating strategy. The VO_2_ array sample fabricated with a 149 μm mesh exhibits good visible transparency (Figure S3f). The ultraviolet–visible–near-infrared (UV–vis–NIR) spectra of the VO_2_-based planar multilayer structure^24^ (marked as “Control group”) and VO_2_ array-based F–P resonator with 57 and 149 μm mesh opening sizes in different application scenarios are shown in Figure 1f. For the winter scenario, the samples show high *T*_lum/sol/NIR_ values up to 50.7%, 48.0%, and 48.0%, respectively. While for the summer scenario, the *T*_lum/sol/NIR_ of the samples largely decreases to minimum values of 8.1%, 7.3%, and 6.4%, respectively. Compared with the control group, the VO_2_ array-based F–P resonator shows greatly enhanced Δ*T*_sol_ and Δ*T*_NIR_ performance (up to 34.6% vs 7.7% and 33.4% vs 12.0%, respectively). The observation demonstrates that the VO_2_ array in the structure can effectively modulate sunlight in response to θ variation and temperature. The VO_2_ arrays show size-related modulation performance (Figures 1g and S8): For instance, the arrays fabricated with a 57 μm mesh achieves a *T*_lum_ of 49.5% and Δ*T*_sol/NIR_ values of 34.6% and 33.2%, respectively. When the mesh opening increases to 209 μm, these values decrease to a *T*_lum_ of 31.0% and Δ*T*_sol/NIR_ of 22.7% and 20.7%, respectively. The ε_broadband_ modulation behavior of the VO_2_ array-based F–P resonator is visualized via IR image (Figure 1h). At a perpendicular θ and LT, the IR image of the sample appears dark, indicating a low ε_broadband_. On the other hand, by tilting the θ and elevating the temperature, the sample exhibits an increased ε_broadband_ visualized by a brighter color in the IR image. Furthermore, the ε_broadband_ modulation ability of the samples was systematically evaluated against their mesh opening sizes (Figure 1i,j). Low ε_broadband_ (∼0.3) is consistently observed in winter scenarios across samples with all mesh sizes and the control group (Figure 1i). While in the summer scenario, the ε_broadband_ values of samples and the control group increase abruptly. By increasing the size of the mesh opening from 57 to 209 μm, the Δε_broadband_ gradually increases from 0.36 to 0.48 (Figure 1j). The change in solar modulation and ε_broadband_ modulation caused by the different mesh opening sizes demonstrates the capability to customize the heat management modulation ability of the window.

**Figure 1:**
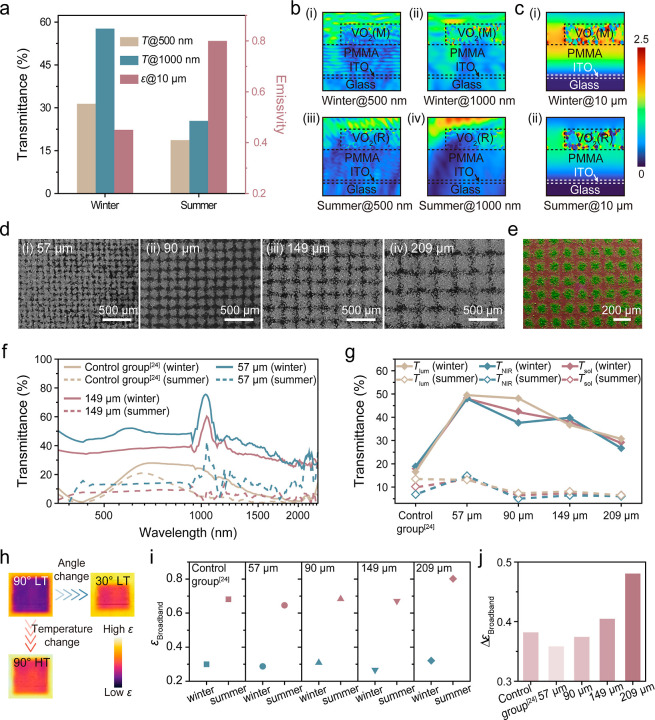
(a) Numerical results of the FDTD simulation for the prototype’s transmittance and emissivity modulation performance under winter (θ: 90°, temperature: 30 °C) and summer scenarios (θ: 30°, temperature: 90 °C). (b) Simulated propagation of light at different wavelengths under seasonal conditions: (i) winter, 500 nm; (ii) winter, 1000 nm; (iii) summer, 500 nm; and (iv) summer, 1000 nm. (c) Simulated propagation of 10 μm wavelength light through the structure under (i) winter and (ii) summer scenarios. Electric field distribution maps, where the horizontal (*x*-axis) represents the lateral dimension and the vertical (*z*-axis) indicates depth. The color scale represents relative electric field intensity, with colors closer to red indicating higher intensity and those closer to blue indicating lower intensity. (d) Microstructure of VO_2_ array samples fabricated with different mesh opening sizes ((i) 57 μm, (ii) 90 μm, (iii) 149 μm, and (iv) 209 μm). (e) Overall EDS mapping image for the VO_2_ array sample. (f) UV–vis–NIR spectra of the control group;^24^ samples with the mesh sizes of 57 μm and 149 μm for winter and summer application scenarios. (g) Optical properties of the control group^24^ and samples with different mesh opening sizes in summer and winter scenarios. (h) IR photos of VO_2_ array samples at different θ and temperatures. (i) Emissivity according to winter and summer scenarios of the control group^24^ and samples fabricated with different mesh opening sizes. (j) Values of Δε_broadband_ for the control group^24^ and samples fabricated with different mesh opening sizes.

### Scalable Smart Windows with Angular–Thermal-Dependent Dual-Modulation

With the scalable solution-based fabrication method, the season-dependent dual-modulation smart window has promising potential for industrial large-scale production, as showcased by a highly uniform window prototype of 100 cm^2^ (Figure 2a). With balanced thermochromic properties and Δε_broadband_, the mesh with a 149 μm opening size is selected for fabrication of the large-scale smart window (Figure S9). Figure 2b reveals the optical spectrum of the large-scale smart window, where UV–vis–NIR spectra show promising winter transmittances (*T*_lum_: 39.6%, *T*_sol_: 37.6%, *T*_NIR_: 36.4%) and low summer transmittances (*T*_lum_: 7.9%, *T*_sol_: 6.7%, *T*_NIR_: 5.8%) that meet the recommendation of energy standard of American Society of Heating, Refrigerating, and Air-Conditioning Engineers (ASHRAE)^27^ and reflect the window’s robust seasonal solar modulation performance (Δ*T*_sol_: 30.9% and Δ*T*_NIR_: 30.6%). Meanwhile, the large-scale smart window shows a promising Δε_broadband_ value of 0.4. The IR photos of the large-scale smart window (Figure 2c) further confirm its promising thermal radiation modulation ability. Figure 2d­(i–iv) illustrates contour maps of transmittance and emissivity across a range of wavelengths as a function of θ. At both LT and HT, transmittance gradually decreases with decreasing θ (Figure 2d­(i,ii)). Notably, at HT, transmittance values are generally lower than those observed at LT, indicated by increased blue-to-purple regions across the wavelength range. In contrast, the emissivity increases with decreasing θ at both temperatures (Figure 2d­(iii,iv)). Specifically, at HT, emissivity values are markedly higher than those at LT across the wavelength range, as indicated by the increased prevalence of near-red regions in the contour maps. The angle- and temperature-dependent dual-modulation ability of the prototype was further validated under varying θ. Figures 2e and S15 present the transmittance and emissivity modulation performance of the prototype at various θ ranging from 0° to 90° under both LT and HT. The prototype demonstrates robust angle-dependent transmittance modulation performance (Figures 2e­(i,ii) and S15). The prototype achieves maximum *T*_lum_, *T*_sol_, and *T*_NIR_ values of approximately 40% at a θ of 90° with LT. As the θ decreases toward conditions typical of summer scenarios, *T*_lum_, *T*_sol_, and *T*_NIR_ gradually diminish. Specifically, at an intermediate θ of 45° and a HT, notable reductions in *T*_lum_, *T*_sol_, and *T*_NIR_ to approximately 10% are observed. At the lowest θ tested (10°), *T*_lum_, *T*_sol_, and *T*_NIR_ further decrease to minimal values of 0.9%, 4.5%, and 11.2%, respectively, confirming the effective angle-dependent transmittance modulation. Concurrently, the prototype exhibits an angle-dependent emissivity modulation performance (Figure 2e­(iii)). Specifically, it shows a low broadband IR emissivity (ε_broadband_ ≈ 0.30) at a θ of 90° under LT conditions. Meanwhile, as θ decreases to 45° at HT, ε_broadband_ markedly increases to 0.64, signifying enhanced thermal radiation directed toward the sky. At a θ of 10° under HT conditions, the prototype reaches a peak ε_broadband_ value of 0.74. These results underscore the prototype’s effectiveness in adapting to varying θ and temperatures, making it particularly suitable for regions with distinct seasonal variations, such as midlatitude climates in the northern hemisphere.

**Figure 2:**
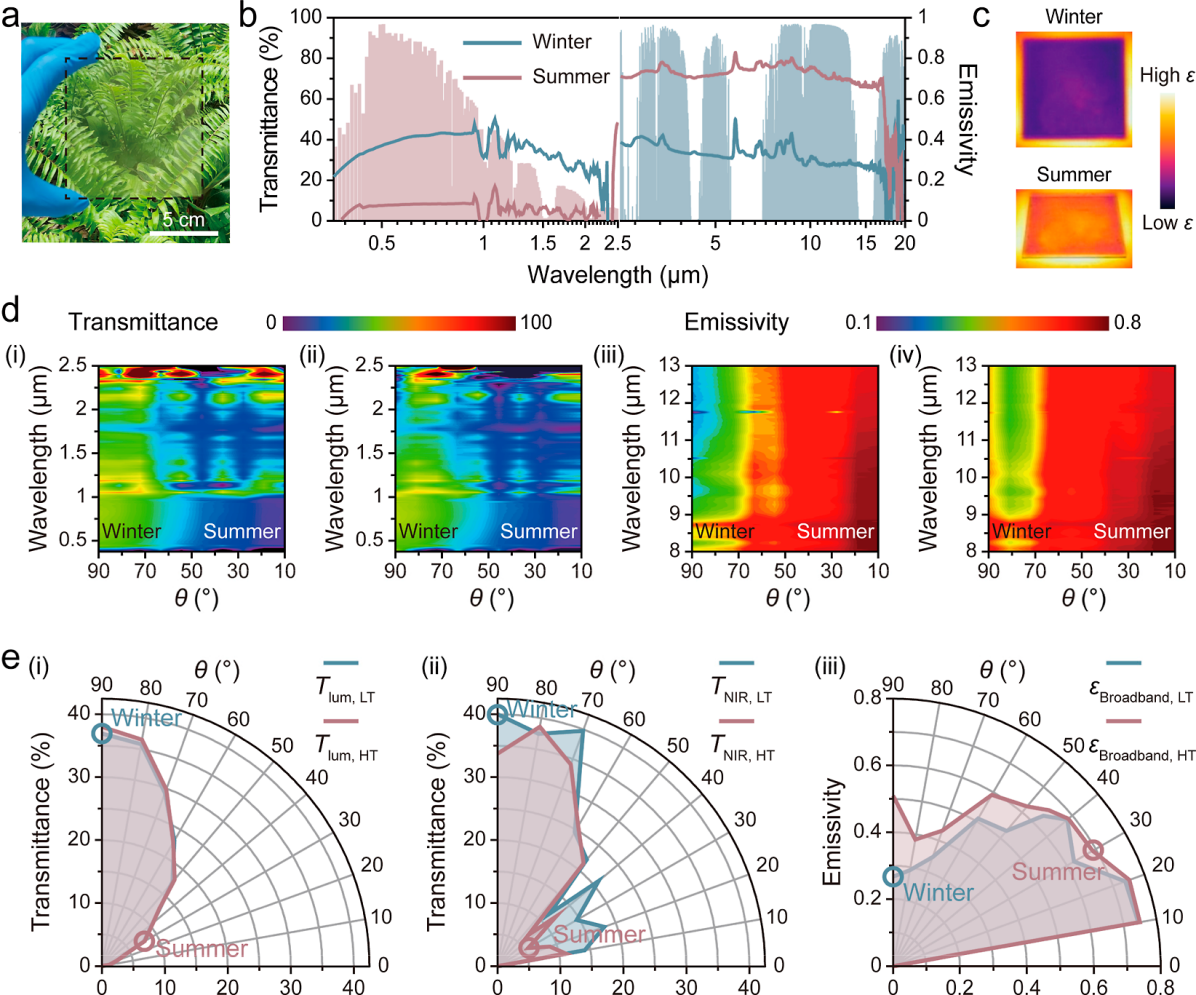
(a) Photo of a large-scale season-dependent smart window with the size of 100 cm^2^. (b) UV–vis–NIR and emissivity spectra of the window in the winter and summer application scenarios against a normalized AM1.5 global solar spectrum (red shadow) and atmospheric transmittance window (blue shadow), respectively. (c) IR images of the smart window for winter (above) and summer (below) application scenarios. (d) Angle- and wavelength-resolved transmittance maps at (i) 30 °C and (ii) 90 °C and emissivity maps at (iii) 30 °C and (iv) 90 °C. In each contour plot, the *x*-axis represents the θ (°), the *y*-axis denotes the wavelength (μm), and the color scale indicates (i,ii) transmittance (%) and (iii,iv) emissivity intensity—where red corresponds to higher values and blue to lower values. (e) Various angle-dependent modulation performances of the prototype at 30 and 90 °C: (i) *T*_lum_, (ii) *T*_NIR_, and (iii) ε_broadband_.

### Color Rendering and Solar Management Performance Balance of the Season-Responsive Smart Window

We further calculated the color rendering index (CRI) and correlated color temperature (CCT) of the fabricated large-scale smart window. The smart window exhibits superior average color rendering indices (Ra), significantly outperforming conventional thermochromic (TC) windows in both winter and summer (98.0 vs 93.4 and 97.6 vs 88.2, respectively; Figure 3a) while maintaining CCTs of approximately 5000 K close to the sunlight, demonstrating its colorless nature (Figure 3b­(i)). In contrast to the yellowish tint continuous VO_2_ thin film^24^ (i.e., the control group), the VO_2_ array-based smart window shows a nearly colorless tint (Figure 3b­(ii)). The CRI and CCT values of the smart window are compared with electrochromic (EC), photochromic (PC),^28^ and conventional VO_2_ TC windows^24^ (the control group in Figure 3b­(ii)) in Figure 3c,d, respectively. With the highest Ra among the samples, the light transmitted through the season-dependent dual-modulation smart window has the best color rendering performance (Figure 3c). On the other hand, compared with the close-to-the-sunlight CCTs of the season-dependent dual-modulation smart window that are kept around 5000 K, the colored EC and PC windows have high CCTs (17,500 and 7500 K, respectively) that are far from the daylight (Figure 3d). Moreover, due to the yellowish color of VO_2_, the conventional VO_2_-based TC window has low CCTs (around ∼3000 K), in both winter and summer scenarios. Both CRI and CCT values suggest that the developed smart window is a promising candidate as glazing to fulfill the aesthetic demand of the users. The performance of the designed season-dependent dual-modulation smart window in the perspective of *T*_lum_, Δ*T*_sol_, and Δε_broadband_ is compared with the previously reported VO_2_-based smart windows such as continuous VO_2_ film-based F–P resonator,^24^ 3D-printed VO_2_ grating,^25^ tungsten (W)-doped VO_2_-silica (SiO_2_) core–shell structure,^29^ and VO_2_-based porous coating.^30^ Compared with these designs, our proposed design possesses balanced performance in all three performance indices (Figure 3e), which demonstrates its promising solar and heat management capabilities.

**Figure 3:**
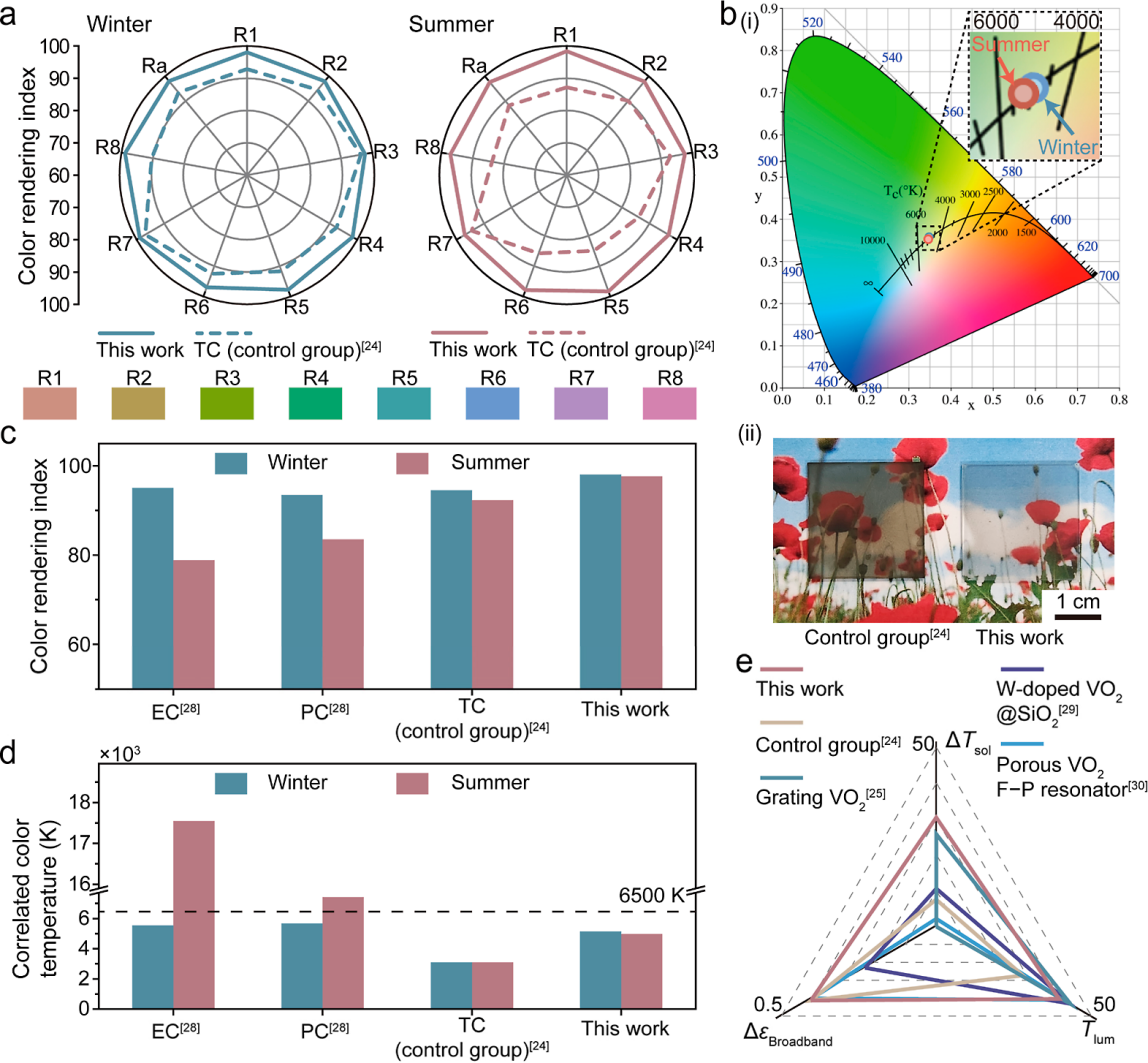
(a) CRI of the season-dependent smart window and TC smart window^24^ for winter and summer. Each label from R1 to R8 corresponds to a specific color. (b) (i) CCT of a season-dependent smart window and (ii) photos of a conventional VO_2_ smart window (Control group)^24^ and this work. The background image adapted from Yaroslav Danylchenko, Freepik. (c) CRI and (d) CCT comparison bar chart (blue: winter, red: summer) for EC, PC, TC smart window, and this work. Data of EC and PC windows were retrieved from ref 28. Data of TC smart window was obtained by measuring the control group.^24^ (e) Radar chart comparing the performance of this study with previously reported results on VO_2_-based smart windows,^24,25,29,30^ regarding the performance evaluation parameters.

### Energy Conservation and Daylight Illumination in Season-Responsive Smart Windows

To investigate the energy-saving performance of the fabricated season-dependent dual-modulation smart window in actual-sized building, the annual energy-saving performance of the season-dependent dual-modulation smart window, low-E glass, and two state-of-the-art samples, namely, planar control sample with thermal radiation modulation^24^ and angle-dependent thermochromic grating structure without thermal radiation modulation,^25^ is compared with the baseline of clear glass in cities with significant seasonal θ variability and ambient temperature difference—Seattle, London, and Seoul. In this actual-sized building energy consumption simulation, a single-story, small building was used as a building model (Figure S2). In all three cities, the proposed smart window outperforms low-E glass and the other two state-of-the-art samples with regard to energy savings (Figures 4a and S17). It shows energy-saving improvements up to 10.6% compared to low-E glass and 6.0% energy-saving improvements compared to the planar control sample. Figure 4b illustrates the monthly energy-savings of the season-dependent dual-modulation smart window, low-E glass, and the planar control sample in each city with clear glass as a baseline. Across all cities, the season-dependent dual-modulation smart window shows the best energy-saving performance among the samples. The observation of the performance differences between the season-dependent dual-modulation smart window and the planar control sample highlights the importance of considering season-accompanied θ variation. Figure 4c presents the winter and summer daylight illuminance of clear glass, low-E glass, season-dependent dual-modulation smart windows, and the planner control sample in the three cities. With a daylight illuminance of more than 2000 lux in winter and summer, the glare accompanied by clear glass and low-E glass may potentially cause visual discomfort.^31^ On the other hand, the season-dependent dual-modulation smart window shows effective daylight illuminance in the range of 100–2000 lux. These results show that the proposed design of the periodic VO_2_ array-based F–P resonator for the smart window has great potential in energy-saving and sunlight management.

**Figure 4:**
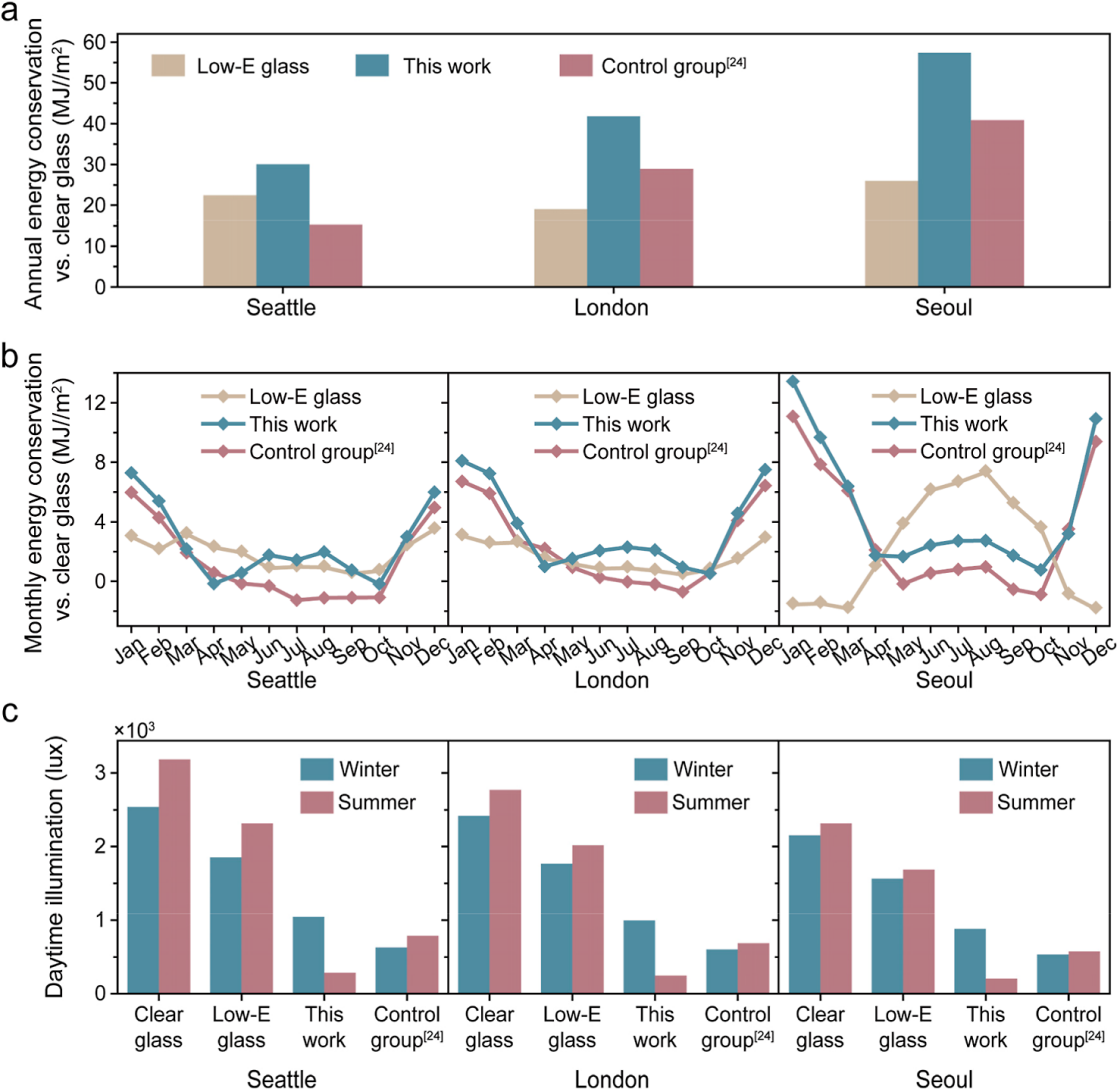
(a) Annual energy-savings of commercial low-E glass, season-dependent dual-modulation smart window designed in this paper, and planar control sample^24^ in Seattle, London, and Seoul with clear glass as baseline. (b) Monthly energy-saving for commercial low-E glass, the season-dependent smart window, and the planar control sample^24^ in Seattle, London, and Seoul with clear glass as baseline. (c) Average winter and summer daylight illumination of clear glass, low-E glass, season-dependent smart window, and the planar control sample^24^ in Seattle, London, and Seoul (blue: winter, red: summer).

## Conclusion

In this study, we designed a dynamic metasurface that gives a high spectral selectivity and enhanced modulation capabilities. An angle/ambient temperature responsive solar/thermal radiation dual-modulation smart window is prototyped that consists of a periodic VO_2_ array-based F–P resonator via an industrial-compatible mesh printing and spray-coating process. With promising luminous transmittance (36.8%), solar modulation ability (30.8%), and broadband IR emissivity modulation properties (0.4), the season-dependent dual-modulation smart window shows a promising energy-saving performance of up to 10.6% compared to low-E glass and 6.0% compared to the state-of-the-art in the regions with the distinct season. In addition, the developed smart window shows promising daylight illumination across the season compared with the planar surface, and it outperforms conventional VO_2_-based thermochromic smart windows in terms of color rendering index and correlated color temperature. In addition, the spray-coating and mesh printing process used for the smart window provide the potential for large-scale industrial production. This proposed design rule by integration of a smart metasurface opens a new avenue to fabricate highly selective spectral modulated devices, which could have wide applications not limited to seasonal-dependent dual-modulation smart window.

## Methods

### Materials

Glass with a single-sided ITO coating (provided by Wintek Technology) measuring 2.5 cm × 2.5 cm for performance optimization and 10 cm × 10 cm for the large-scale prototype, ethanol (95%, Aik Moh), acetone (95%, Aik Moh), PMMA (*M*_w_ ∼ 120,000, Sigma-Aldrich), VO_2_ nanoparticles (VO_2_ NPs, All-India Metal Corp.), and mesh (provided by SEFAR Singapore, made with polyethylene terephthalate) were used as received without further purification.

### Substrate Preparation

Glass with a single-sided ITO coating was washed with ethanol and used as a substrate. 500 μL of PMMA solution prepared by dissolving 0.5 g of PMMA in 10 mL of acetone was spin-coated on ITO glass with a variable spin speed of 500, 1000, 1500, 2000, 2500, and 3000 rpm via a spin coater (POLOS SPIN150i). The samples with different spin speeds were then used to investigate the effect of the PMMA spin-coating speed on the samples’ solar/emissivity modulation ability.

### VO_2_ Ink Preparation

VO_2_ NPs were used as a material for producing VO_2_ ink for spray-coating without any additional purification process. VO_2_ NPs and 0.05 g of PMMA were dispersed into 5 mL of acetone and then sonicated in iced water for 2 h to prepare the VO_2_-contained ink for subsequent spray-coating. The prepared VO_2_ ink was filtered through a syringe filter with a pore size of 1.2 μm to remove the agglomerated particles. The concentration of VO_2_ in the ink was varied to investigate the impact of the VO_2_ concentration on the modulation ability of samples.

### Mesh Printing and Spray-Coating

To form the VO_2_ array, a mesh was attached to the PMMA-coated substrate and spray-coating was conducted with a spray gun (Eidolon spray gun, model JP-10). During spray-coating, the adjusting pin screw and nozzle size were 1.5 mm and 0.3 mm, respectively. To improve the uniformity of coating, the substrate was preheated at 60 °C for 10 min before spray-coating, and the heating was maintained during spray-coating. Factors such as mesh opening size, spray-coating distance, and coating time were systematically tuned and are summarized in Table S1.

### Large-Scale Sample Preparation

Glass with a single-sided ITO coating measuring 10 cm × 10 cm (100 cm^2^) was washed with ethanol and used as a substrate. 8 mL of PMMA solution prepared by dissolving 0.5 g of PMMA in 10 mL of acetone was spin-coated on ITO glass. Subsequently, the large-area mesh with an opening size of 149 μm was attached to the PMMA-coated substrate, and the substrate was sufficiently heated to 60 °C. The VO_2_ ink prepared previously was spray-coated while maintaining the elevated temperature of the substrate.

### Characterization

The crystal structure and particle size of VO_2_ NPs were characterized using transmission electron microscopy (TEM, JEOL 2010) and FESEM, JEOL JSM-7800F PRIME. Additionally, the microstructure of the spray-coated VO_2_ array was analyzed using SEM. Cross-sectional profiles were obtained from optical microscope images acquired using an Olympus BX61 motorized microscope. The average particle size and average size of the coated VO_2_ array were determined using standard software (IMAGE J). To confirm the formation of the VO_2_ array in the mesh coating, it was estimated using EDS. The crystal structure of VO_2_ was analyzed qualitatively using X-ray diffraction (XRD), Bruker D8 Advance using Cu Kα radiation (λ = 0.154 nm) in the 2θ range of 20–70°. Bonding information on VO_2_ NP was confirmed through Fourier transform infrared (FTIR, PerkinElmer Frontier). The solar modulation of the manufactured angle-dependent dual-modulation smart window according to temperature was analyzed using a UV–vis–NIR high-sensitivity spectrometer (Avantes AvaSpec-ULS2048L StarLine Versatile Fiber-optic Spectrometer and AvaSpecNIR256-2.5-HSC-EVO) with a temperature controlling stage (Linkam, PE120) attached; and the spot size for the spectrometer was 0.5 cm × 0.5 cm. ε_broadband_ of the samples was measured by a dual-band emissivity measurement instrument (IR-2, Shanghai Chengbo Photoelectric Technology) with a heating stage. The ε_broadband_ values of five points in the sample were recorded, and the average ε_broadband_ value was calculated. Emissivity curve collection was conducted using a Parkin Elmer Frontier spectrometer with an integrated sphere attached. The spot size of the FTIR spectrometer was 2 cm × 2 cm. Here, the sample temperature was controlled by a self-designed heating plate, and a self-made blackout box was used to analyze a tilted sample. IR images were captured with an IR camera (FLIR E4) according to the temperature and angle of the manufactured VO_2_ smart window.

## Supplementary Material



## Notes

Transmittance, transmittance modulation, and emissivity modulation calculations; building energy-saving and daylight illumination simulation details via EnergyPlus; CRI and CCT calculations; daily solar radiation data for northern hemisphere midlatitudes between December and June; morphological, structural, and compositional characterization of VO_2_ NPs (TEM, SEM, XRD, FTIR), including EDS mapping of the VO_2_ array; FDTD simulation parameters and setup; emissivity modulation performance analysis according to VO_2_ concentration, spray-coating distance, coating time, and spin-coating speed of the PMMA layer; optical and emissivity modulation performance analysis under various mesh opening sizes, including photographs, IR thermal images, and UV–vis–NIR and broadband emissivity spectra; comparative optical and thermal radiation performance of the control group (stacked PMMA/ITO on glass); optical modulation response of the periodic VO_2_ array under varying solar zenith angles; CCT evaluation of continuous VO_2_ film-based control sample; comparative energy-saving performance analysis of a grating structure and the season-dependent dual-modulation smart window; parameter table for mesh printing and spray-coating conditions; building model specifications for energy simulation; tables summarizing the optical and thermal properties of the fabricated dual-modulation smart window, grating structure, planar control sample, commercial low-E glass, and clear glass; summary table comparing this work to previously reported VO_2_-based smart windows; and supporting reference list included (PDF)
