# Supplementary material for: Angular-Dependent Energy-Saving Smart Windows
Source: ACS Nano. 2025 Sep 15;19(38):34429–37. doi: 10.1021/acsnano.5c13103 (PMC12490002; doi:10.1021/acsnano.5c13103)
Supplement: Supplementary file 1 [file nn5c13103_si_001.pdf]

Supporting Information

Angular Dependent Energy Saving Smart Windows

Keunhyuk Ryu[a,b], Guanya Wang[b], Vijay Shankar Sridharan[a], Shancheng Wang[b,*], ZhiLi Dong[a,*] Shuang Zhang[c], and Yi Long[b,*]

[a] School of Materials Science and Engineering, Nanyang Technological University, 639798, Singapore.

[b] Department of Electronic Engineering, The Chinese University of Hong Kong, New Territories, 999077, Hong Kong SAR, China.

[c] New Cornerstone Science Laboratory, Department of Physics, University of Hong Kong, 999077, Hong Kong, China.

* Corresponding authors.

E-mail addresses: yilong@cuhk.edu.hk (Y. Long), ZLDong@ntu.edu.sg (Z. L. Dong), shanchengwang@cuhk.edu.hk (S. Wang)

Calculation of angle and temperature-dependent transmittance and solar modulation ability

The luminous transmittance (T_{lum}), solar transmittance (T_{sol}) and near-infrared transmittance (T_{NIR}) were calculated with the formula:

$$T_{lum/sol/NIR} = \frac{\int \varphi_{lum/sol/NIR}\, T(\lambda) d\,\lambda}{\int \varphi_{lum/sol/NIR} d\,\lambda}$$

The $T(\lambda)$ is the spectral transmittance (360 nm-780 nm for T_{lum}, 360 nm-2500 nm for T_{sol}, and 790 nm-2500 nm for T_{NIR}). $\varphi_{lum}(\lambda)$ is the standard luminous efficiency function of photopic vision for the wavelength of 380-780 nm.[1] $\varphi_{sol}(\lambda)$ and $\varphi_{NIR}(\lambda)$ are the solar/NIR irradiance spectra for air mass 1.5 (corresponding to the sun standing 37° above the horizon with 1.5-atmosphere thickness, corresponds to a solar zenith angle of 48.2°) as wavelength (λ) functions.[1, 2] $T(\lambda)$ is the transmittance of light at wavelength λ. The luminous modulation (ΔT_{lum}), solar modulation (ΔT_{sol}), and NIR modulation (ΔT_{NIR}) can be calculated by:

$$\Delta T_{lum/sol/NIR} = T_{lum/sol/NIR}\,(30\,°C) - T_{lum/sol/NIR}\,(90\,°C)$$

Calculation of angle and temperature-dependent broadband infrared (broadband IR) emissivity

Broadband IR emissivity modulation ability ($\Delta\varepsilon_{Broadband}$) was calculated with the following formula:

$$\Delta\varepsilon_{\text{Broadband}} = \varepsilon_{\text{Broadband-H}} - \varepsilon_{\text{Broadband-L}}$$

$\varepsilon_{Broadband\text{-}H}$ refers to the integrated broadband emissivity of the sample measured at high temperature (HT, 90 °C), and $\varepsilon_{Broadband\text{-}L}$ refers to the integrated broadband emissivity of the sample measured at low temperature (LT, 30 °C). The $\varepsilon_{Broadband}$ spectral curve was plotted

according to Kirchhoff's law of thermal radiation: $\varepsilon(\lambda) = A(\lambda) = 1-T(\lambda)-R(\lambda)$.[3, 4] Where $R(\lambda)$ is the spectral broadband reflectance and $T(\lambda)$ is the spectral broadband transmittance measured with a Fourier transform infrared spectroscopy (FTIR) spectrometer equipped with an integrating sphere.

Actual building energy consumption simulation and daylight illumination simulation via EnergyPlus

A single-story small house building model was used to investigate the impact of T_{sol} and $\varepsilon_{Broadband}$ on building energy consumption. Energy consumption was simulated using EnergyPlus by applying an "ideal-load-air-system" provided by a district heating/cooling source. These results closely represent the T_{sol} and $\varepsilon_{Broadband}$ characteristics for space heating loads. In the case of daylight illumination, the value for summer was calculated as the average daylight illumination from June to August, and the value for winter was calculated as the average daylight illumination of the month (December, January, and February). The building dimensions are 8 m(L) × 6 m(W) × 2.7 m(H) (Figure S1). The insulation meets the requirements of ASHRAE standard 90.1.[5] In the model, the total window glass area of the glass system is 24 m^2, with a window-to-wall ratio of 27.8%, distributed over four walls. Two people and the electricity appliance with a total power of 6 W/m^2 were used as internal load. Building specifications for energy-saving simulations are summarized in Table S2. Cities with clear seasonal changes in solar elevation were selected for simulation: Seattle, WA, USA, London, UK, and Seoul, Korea. T_{lum}, T_{sol}, and $\varepsilon_{Broadband\text{-}Front}$ were collected for each window type (clear glass, low-emissivity (low-E) glass, angle-dependent thermochromic grating structure without thermal radiation modulation, planar control sample with thermal radiation modulation and season-dependent dual-modulation smart window), with $\varepsilon_{Broadband\text{-}Back}$ fixed at 0.84 and all glass was considered single-glazed windows. The optical properties of clear glass were obtained from the EnergyPlus database. CNG Online Sun-E™ was used as the low-E glass. The detailed optical properties of each window are summarized in Table S3.

Color rendering index (CRI) and correlated color temperature (CCT) calculations

The CRI and CCT of different samples were calculated based on the method discussed in reference.[6, 7]

CCT calculations

For the CCT calculations, the tristimulus values X, Y, and Z for the light transmitted through the samples were calculated with the formulae:

$$X = \sum_{380\,nm}^{780\,nm} D_{65}(\lambda)\tau(\lambda)\bar{x}(\lambda)\Delta\lambda$$

$$Y = \sum_{380\,nm}^{780\,nm} D_{65}(\lambda)\tau(\lambda)\bar{y}(\lambda)\Delta\lambda$$

$$Z = \sum_{380\,nm}^{780\,nm} D_{65}(\lambda)\tau(\lambda)\bar{z}(\lambda)\Delta\lambda$$

Where: $D_{65}(\lambda)$ is the spectral power distribution for the D65 standard light source.[8] $\tau(\lambda)$ is the spectral transmittance value measured by the spectrometer. $\bar{x}(\lambda)$, $\bar{y}(\lambda)$, and $\bar{z}(\lambda)$ are spectral color matching functions of 1931 2°CIE standard observer.[8] The chromaticity coordinates of the samples *(x, y)* were then calculated by:

$$x = \frac{X}{X + Y + Z}$$

and

$$y = \frac{Y}{X + Y + Z}$$

Lastly, the CCT was calculated based on McCamy's equation[9]:

$$CCT = 449n^3 + 3525n^2 + 6823.3n + 5520.33$$

Where n is calculated with the chromaticity coordinates with the formula:

$$n = \frac{x - 0.3320}{0.1858 - y}$$

CRI calculations

For CRI calculation, the tristimulus values of the light transmitted by the samples and reflected by the standard test colours specified by the CIE.[10] The calculation of tristimulus values follows the below formulae:

$$X_i = \sum_{380\,nm}^{780\,nm} D_{65}\,(\lambda)\tau(\lambda)\beta_i(\lambda)\bar{x}(\lambda)\Delta\lambda$$

$$Y_i = \sum_{380\,nm}^{780\,nm} D_{65}\,(\lambda)\tau(\lambda)\beta_i(\lambda)\bar{y}(\lambda)\Delta\lambda$$

$$Z_i = \sum_{380\,nm}^{780\,nm} D_{65}\,(\lambda)\tau(\lambda)\beta_i(\lambda)\bar{z}(\lambda)\Delta\lambda$$

Where $\beta_i(\lambda)$ is the spectral reflectance of standard test colours (i=1 to 8).

The trichromatic coordinates of light transmitted by the sample (u_t, v_t) and light transmitted by the sample and then reflected by the test color ($u_{t,i}$, $v_{t,i}$) were calculated by:

$$u_t = \frac{4X}{X + 15Y + 3Z}$$

$$v_t = \frac{6X}{X + 15Y + 3Z}$$

$$u_{t,i} = \frac{4X_{t,i}}{X_{t,i} + 15Y_{t,i} + 3Z_{t,i}}$$

$$v_{t,i} = \frac{6X_{t,i}}{X_{t,i} + 15Y_{t,i} + 3Z_{t,i}}$$

The trichromatic coordinate correction after distortion by chromatic adaptation is calculated by:

$$u'_{t,i} = \frac{10.872 + 0.8802\frac{c_{t,i}}{c_t} - 8.2544\frac{d_{t,i}}{d_t}}{15.518 + 3.2267\frac{c_{t,i}}{c_t} - 2.0636\frac{d_{t,i}}{d_t}}$$

$$v'_{t,i} = \frac{5.520}{15.518 + 3.2267\frac{c_{t,i}}{c_t} - 2.0636\frac{d_{t,i}}{d_t}}$$

The values c_t, d_t and $c_{t,i}$, $d_{t,i}$ are for transmitted light and light transmitted and then reflected by standard test colours, respectively. They were calculated by formulae:

$$c_t = \frac{4 - u_t - 10v_t}{v_t}$$

$$d_t = \frac{1.708v_t + 0.404 - 1.481u_t}{v_t}$$

$$c_{t,i} = \frac{4 - u_{t,i} - 10v_{t,i}}{v_{t,i}}$$

$$d_{t,i} = \frac{1.708v_{t,i} + 0.404 - 1.481u_{t,i}}{v_{t,i}}$$

The 1964 CIE UCS chromaticity coordinates of the light transmitted and then reflected by standard test colours ($W^*_{t,i}$, $U^*_{t,i}$, $V^*_{t,i}$) were calculated by tristimulus values and trichromatic

coordinate correction as:

$$W^*_{t,i} = 25(\frac{100Y_{t,i}}{Y_t})^{1/3} - 17$$

$$U^*_{t,i} = 13W^*_{t,i}(u'_{t,i} - 0.1978)$$

$$V^*_{t,i} = 13W^*_{t,i}(v'_{t,i} - 0.3133)$$

With comparing the UCS chromaticity coordinates of the light transmitted and then reflected by standard test colours ($W^*_{t,i}$, $U^*_{t,i}$, $V^*_{t,i}$) and the original coordinates of the test colours ($W^*_{r,i}$, $U^*_{r,i}$, $V^*_{r,i}$), the total distortion ΔE_i was calculated by:

$$\Delta E_i = \sqrt{(U^*_{t,i} - U^*_{r,i})^2 + (V^*_{t,i} - V^*_{r,i})^2 + (W^*_{t,i} - W^*_{r,i})^2}$$

Lastly, the colour rendering index R_i for individual standard test colour is calculated by:

$$R_i = 100 - 4.6\Delta E_i$$

While the average colour rendering index R_a is calculated by averaging the colour rendering index for 8 standard text colours.

Solar radiation and solar zenith angle data between December and June

The solar radiation and solar zenith angle (θ) data for mid-latitude regions between December and June were obtained from the National Solar Radiation Database (NSRDB, https://nsrdb.nrel.gov/) and Gaisma (https://www.gaisma.com/en/), respectively. Figure S1

presents the representative solar radiation intensity profiles for selected mid-latitude cities. The grey shadow indicates the solar noon (from 10 a.m. to 2 p.m.).

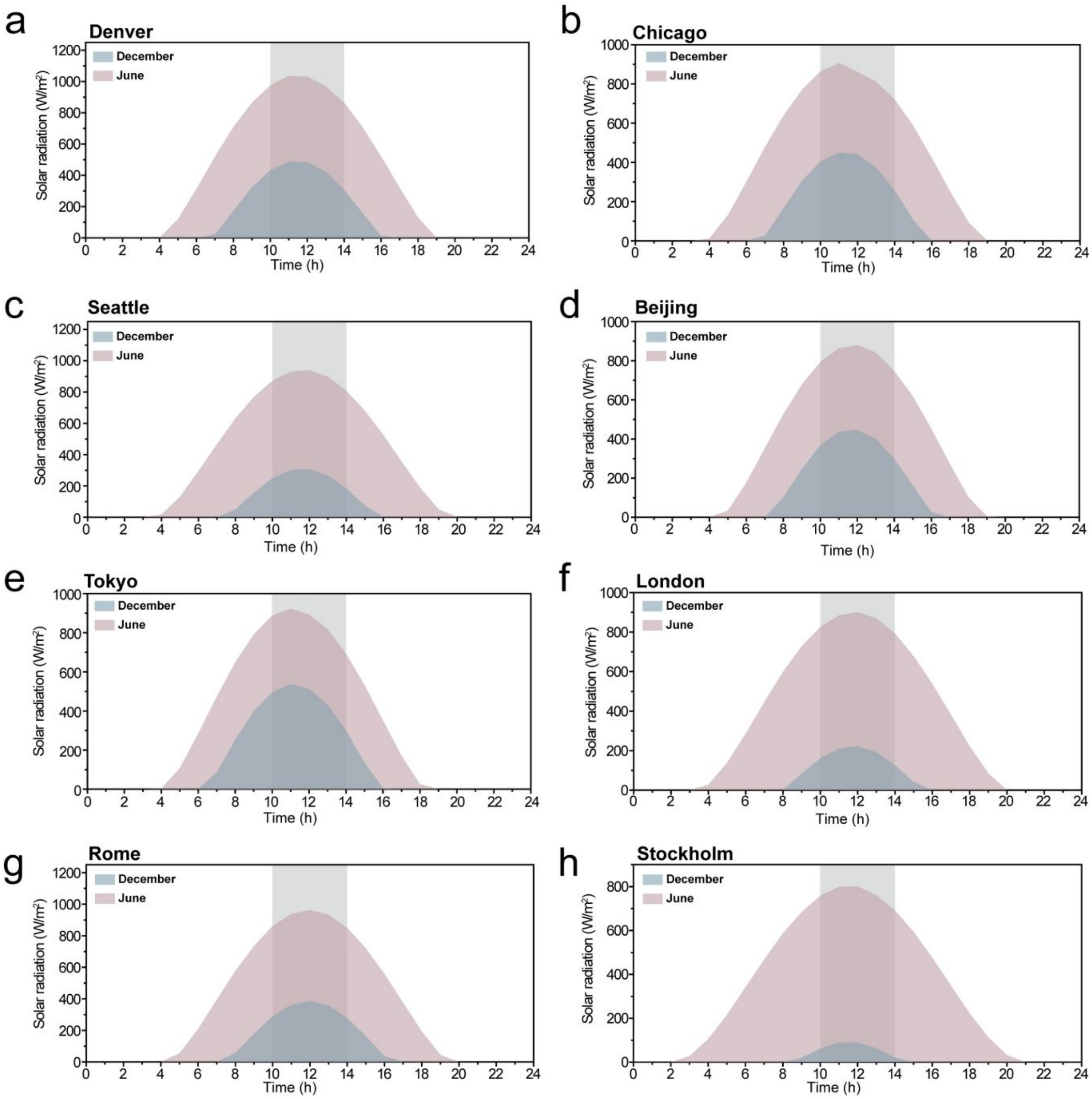

Figure S1. *Solar radiation intensity profiles across different mid-latitude regions: (a) Denver, (b) Chicago, (c) Seattle, (d) Beijing, (e) Tokyo, (f) London, (g) Rome, and (h) Stockholm.*

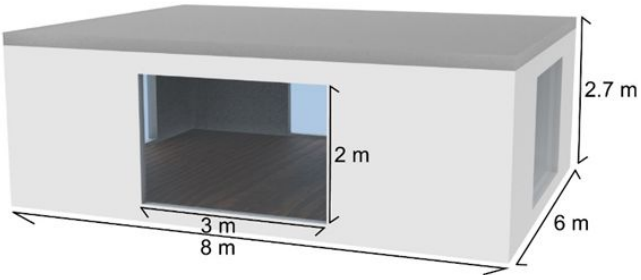

Figure S2. *Building model for energy-saving simulation.*

Morphological, structural, and compositional characterization of vanadium dioxide nanoparticles (VO_2 NPs)

The morphology, crystal structure, and composition of VO_2 NPs used in VO_2 array fabrication were characterized. Under the transmission electron microscopy (TEM), VO_2 NP exhibits a particle size of approximately 150 nm (Figure S3a(i)), and the inter-plane spacings corresponding to the (020) plane and (200) plane of the monoclinic VO_2 (VO_2(M)) phase were identified in the image (Figure S3a(ii)). The scanning electron microscope (SEM) image of VO_2 NPs reveals an average particle size of approximately 180 nm (Figure S3b), agreeing well with the TEM image of VO_2. The X-ray diffraction (XRD) pattern of VO_2 NPs shows the XRD peak from lattice planes (011), (-211), (-212), (-222), and (-213) (Figure S3c), and it matches the characteristic pattern of VO_2(M) (JCPDS index number: 82-0661).[11] As shown in Figure S3d, the absorption peaks located at 530 and 715 cm^{-1} in the FTIR spectrum represent the V-O-V octahedral bending modes and coupled vibration V=O of VO_2 NPs.[11] The energy-dispersive X-ray spectroscopy (EDS) elemental maps the elemental distribution in periodic VO_2 array on the substrate (Figure S3e); green, magenta, and yellow colors represent vanadium (V), silicon (Si), and oxygen (O), respectively. The VO_2 array sample fabricated with 149 μm mesh exhibits good visible transparency (Figure S3f).

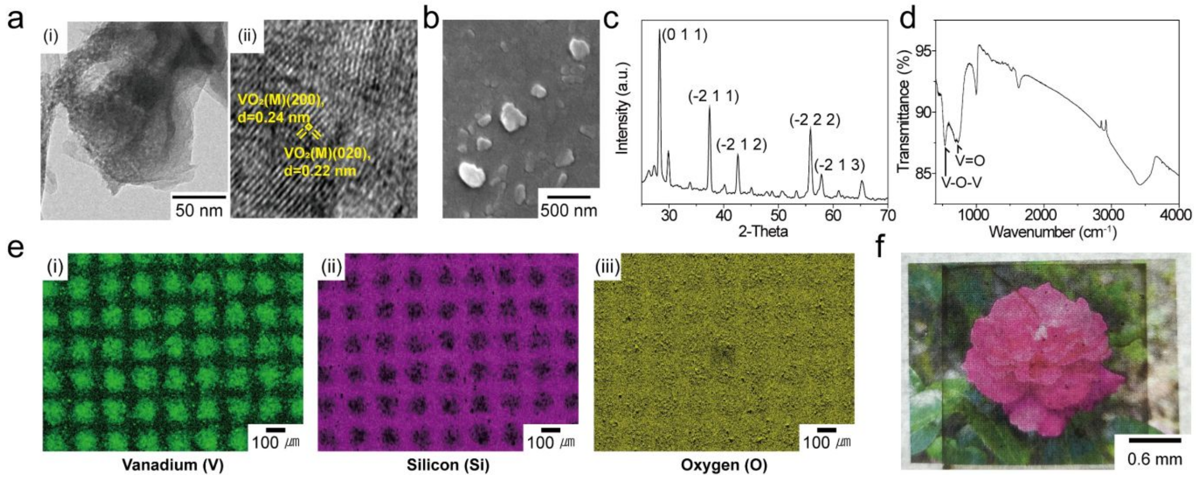

Figure S3. *(a) (i) TEM image of VO_2 NP and (ii) High-resolution TEM image of the VO_2 NP viewing from [001] direction. (b) SEM image, (c) XRD, and (d) FTIR spectrum of VO_2 NPs. (e) EDS mapping images for VO_2 array sample ((i): V, (ii): Si, and (iii): O). (f) Photo of VO_2 array sample fabricated with 149 μm mesh.*

Finite difference time domain (FDTD) simulations detail

FDTD simulations were performed using commercial software (FDTD solution, Lumerical Inc.). The optical constants of VO_2, poly(methyl methacrylate) (PMMA), ITO, and glass were obtained from reference.[12-16] The structure was modelled as a multilayered structure where PMMA and ITO were coated on the glass substrate respectively, and the VO_2 islands composed of NPs were coated above the PMMA layer. The gaps were present between the VO_2 islands. The entire system was suspended in air/vacuum and the incident beam was modelled as a plane wave propagating along the z direction. The θ of the light source was 90° and 30° for the winter and summer scenarios, respectively. Periodic boundary conditions were set for x and y directions, and perfectly matched layer (PML) boundary conditions are set for x, y, and z directions of the simulation regions. Two-dimensional (2D) frequency domain power monitors were placed at fixed z positions below and above the structure to detect the transmitted and reflected beam intensity, and a 2D frequency domain profile monitor was placed at fixed y position to capture the electric field distribution. Mesh sizes of $1 \times 1 \times 1$ nm^3 and $10 \times 10 \times 10$ nm^3 were set on VO_2 islands for the ultraviolet-visible-near infrared (UV-Vis-NIR) and 8-13 μm broadband IR bands, respectively. The other parameters are set as follows: a simulation time of 10000 fs with an auto-shutoff parameter of 10^{-5}, a mesh accuracy of 6, and mesh refinement algorithm set to "conformal variant 1" allowing for a nonuniform mesh over the

FDTD domain.

Periodic VO_2 array modulation abilities control and sample photography fabricated with different mesh opening sizes

Figures S4-S7 show the relationship between modulation abilities and various fabrication parameters of the periodic VO_2 array-based season-dependent smart window such as VO_2 concentration, coating distance, coating time, and spin speed for PMMA layer. Each parameter's impact on $\varepsilon_{Broadband}$ modulation and thermochromic properties is systematically investigated. Figure S4 shows the relationship between $\varepsilon_{Broadband}$ modulation ability and VO_2 concentrations with a fixing spray coating distance of 10 cm, coating time of 25 seconds, and PMMA spin coating speed of 1000 RPM. It identified 0.2625 g of VO_2 as the optimal concentration. Figure S5 depicts how the spray coating distance affects $\varepsilon_{Broadband}$ modulation ability. A distance of 10 cm is identified as the optimal coating distance. Figure S6 presents the effect of spray coating time on $\varepsilon_{Broadband}$ modulation ability. A coating time of 25 seconds is selected as the optimal coating time. Figure S7 illustrates the influence of PMMA spin coating speed on $\varepsilon_{Broadband}$ modulation ability. The highest $\varepsilon_{Broadband}$ modulation ability is shown in the spin coating speed of 1000 RPM. Figure S8 indicates the sample fabricated with 57 μm mesh achieves a ΔT_{lum} of 34.6% with a ΔT_{sol} of 34.6%. While when the mesh opening increases to 209 μm, the ΔT_{lum} and ΔT_{sol} decrease to 24.3% and 22.7%. Figure S9 shows the comprehensive T_{lum}, ΔT_{sol}, and $\Delta\varepsilon_{Broadband}$ ability of the periodic VO_2 array smart window manufactured with various mesh aperture sizes based on the optimal variable combination derived based on $\Delta\varepsilon_{Broadband}$ (VO_2 of 0.2625 g, spray coating distance of 10 cm, spray coating time of 25-second, and PMMA spin coating speed of 1000 RPM). As shown in Figure S9, the sample fabricated with 149 μm mesh opening achieves the most balanced performance (T_{lum}: 36.8%, ΔT_{sol}: 30.8%, and $\Delta\varepsilon_{Broadband}$: 0.4). Figures S10a-d show the photos of the VO_2 arrays fabricated with different sizes of mesh. With the photos of VO_2 array coated samples, it can be observed that all the samples exhibit high transparency.

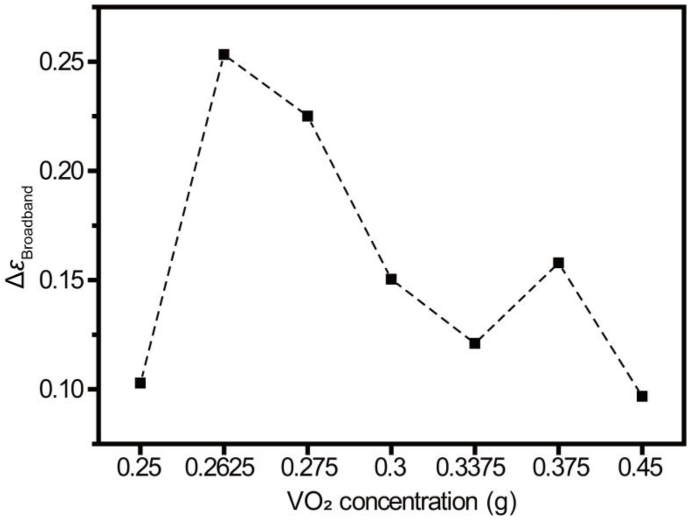

Figure S4. *$\Delta\varepsilon_{Broadband}$ ability according to various VO_2 concentrations with a fixed coating distance of 10 cm, coating time of 25 seconds, and PMMA coating speed of 1000 RPM.*

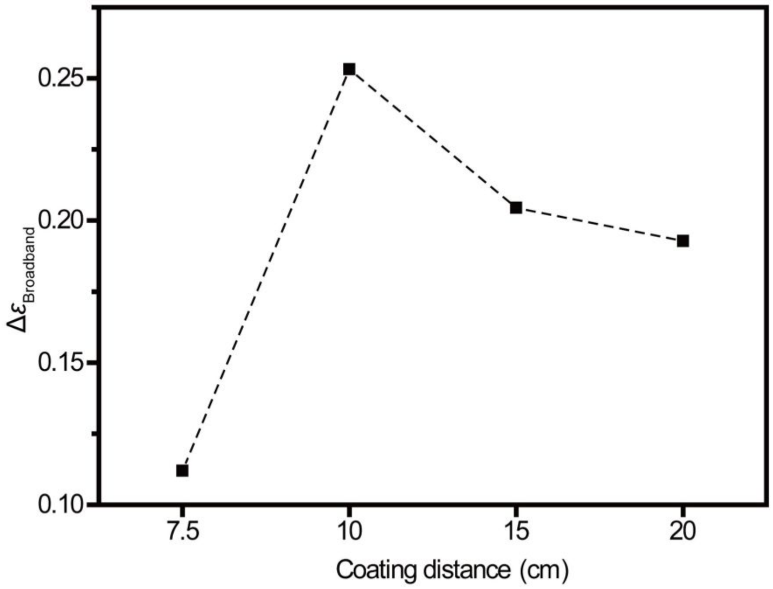

Figure S5. *$\Delta\varepsilon_{Broadband}$ ability according to various coating distances with a fixed VO_2 concentration of 0.2625 g, coating time of 25 seconds, and PMMA coating speed of 1000 RPM.*

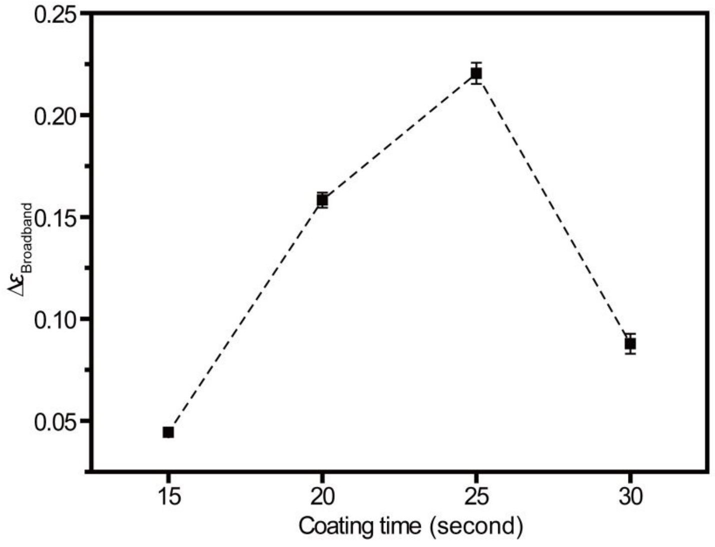

Figure S6. *$\Delta\varepsilon_{Broadband}$ ability according to various coating times with a fixed VO_2 concentration of 0.2625 g, coating distance of 10 cm, and PMMA coating speed of 1000 RPM.*

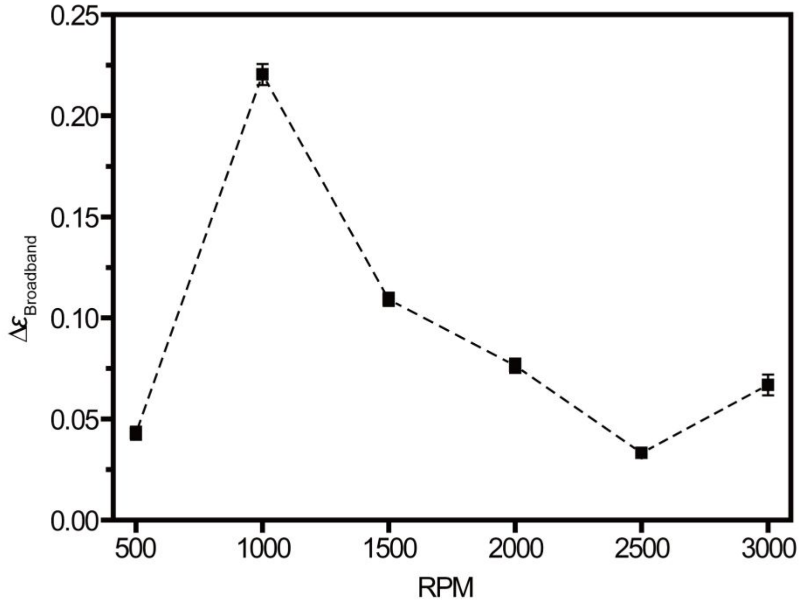

Figure S7. *$\Delta\varepsilon_{Broadband}$ ability according to various PMMA coating RPM with a fixed VO_2 concentration of 0.2625 g, coating distance of 10 cm, and coating time of 25 seconds.*

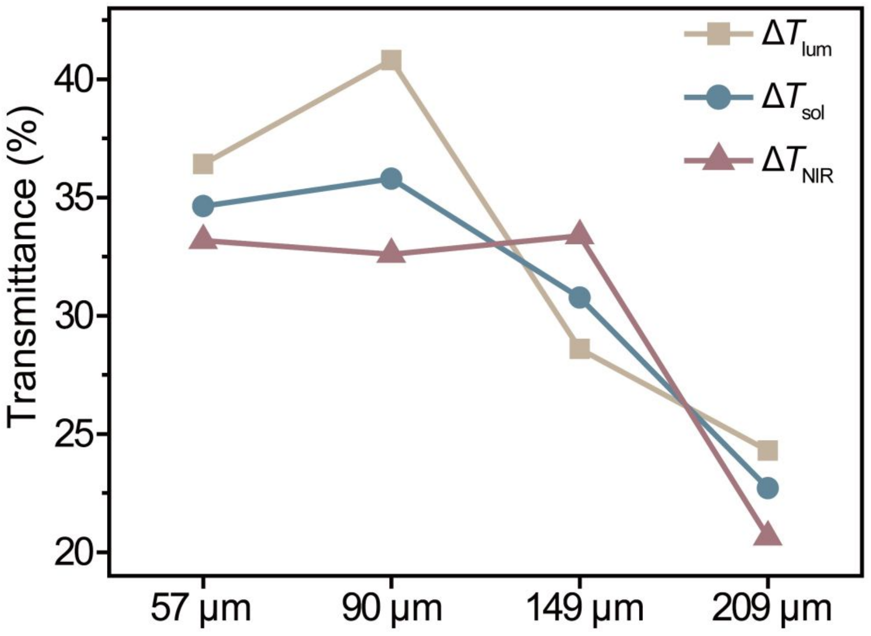

Figure S8. *Thermochromic performance of the samples with different mesh opening sizes.*

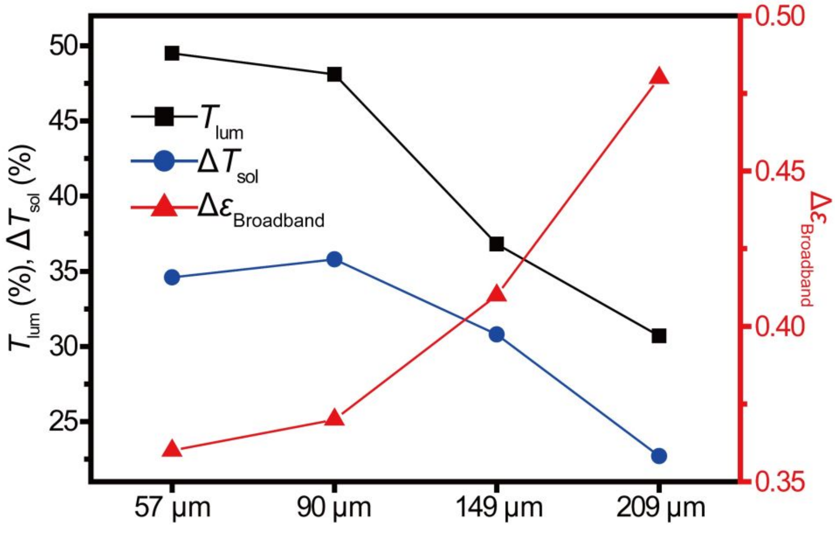

Figure S9. *Optical and $\Delta\varepsilon_{Broadband}$ properties of periodic VO_2 array smart window according to mesh opening size.*

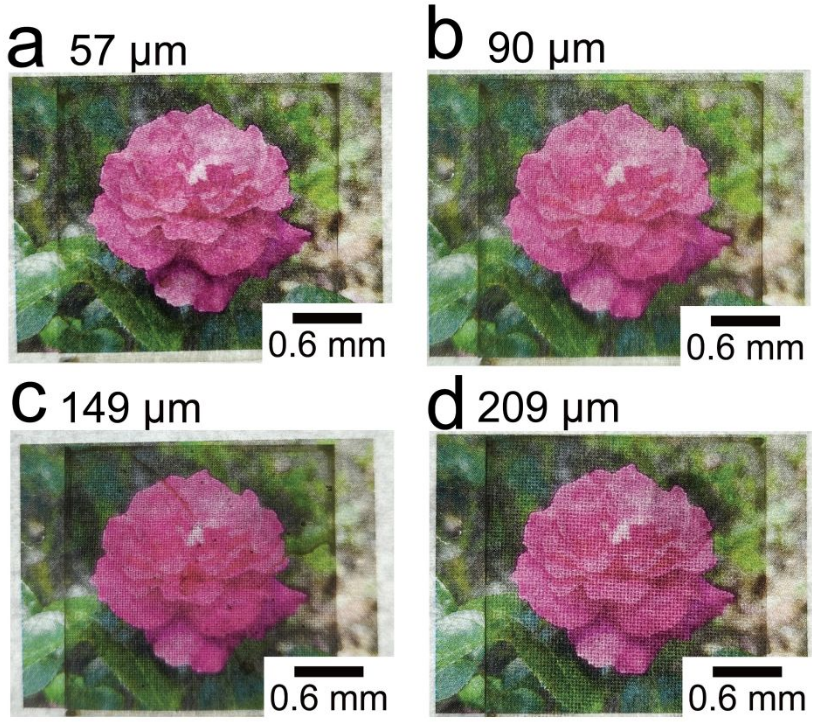

Figure S10. *(a) Photograph of VO_2 array sample with a mesh size of (a) 57 µm, (b) 90 µm, (c) 149 µm, and (d) 209 µm with 25-second spray coating.*

IR photos of angle-dependent dual-modulation smart window fabricated with different mesh opening sizes according to the winter and summer application scenarios

Figure S11 presents the IR photos of the angle-dependent dual-modulation smart window sample fabricated with different mesh opening sizes ((a) 57 μm, (b) 90 μm, (c) 149 μm, and (d) 209 μm). As shown in Figure S11, all samples show dark color in the winter scenario (θ: 90° and temperature: 30 °C), indicating suppressed thermal radiation and lowered $\varepsilon_{\text{Broadband}}$. On the other hand, all samples indicate prompted thermal radiation and increased $\varepsilon_{\text{Broadband}}$ with brighter color in the summer scenario (θ: 30° and temperature: 90 °C) than in the winter scenario.

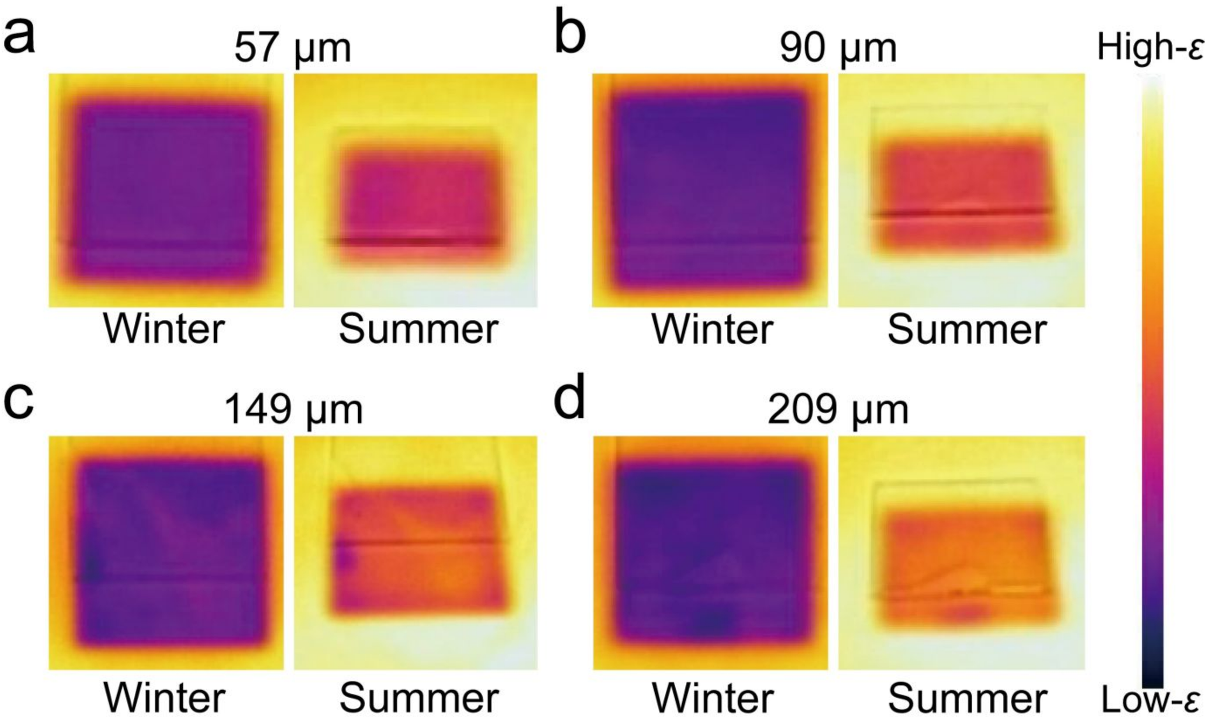

Figure S11. *IR photos of (a) sample fabricated with a 57 μm mesh opening size, (b) sample fabricated with a 90 μm mesh opening size, (c) sample fabricated with a 149 μm mesh opening size, (d) sample fabricated with a 209 μm mesh opening size at winter and summer scenarios: θ of 90° and 30°, and temperature of 30 °C and 90 °C, respectively.*

UV-Vis-NIR and broadband emissivity spectra of the season-dependent dual-modulation smart window manufactured with different mesh opening sizes

Figure S12 presents the UV-Vis-NIR and emissivity spectra of the angle-dependent dual-modulation smart windows fabricated with different mesh opening sizes. The sample fabricated with a 57 μm mesh exhibits relatively high T_{lum} (49.5%), low ΔT_{sol} (34.6%), and $\Delta\varepsilon_{Broadband}$ (0.36) compared to other samples (Figure S12a). The sample fabricated with a 90 μm mesh shows comparable T_{lum} (48.1%) and ΔT_{sol} (35.8%) compared to the sample manufactured with a 57 μm mesh and still lacking $\Delta\varepsilon_{Broadband}$ (0.37) (Figure S12b). The sample fabricated with a 149 μm mesh shows the most balanced thermochromic properties and $\Delta\varepsilon_{Broadband}$ (T_{lum}: 36.8%, ΔT_{sol}: 30.8%, and $\Delta\varepsilon_{Broadband}$: 0.4) (Figure S12c). In contrast, the sample fabricated with a 209 μm mesh demonstrates dramatically decreased T_{lum} (30.7%) and ΔT_{sol} (22.7%), along with an increased $\Delta\varepsilon_{Broadband}$ (0.48) (Figure S12d).

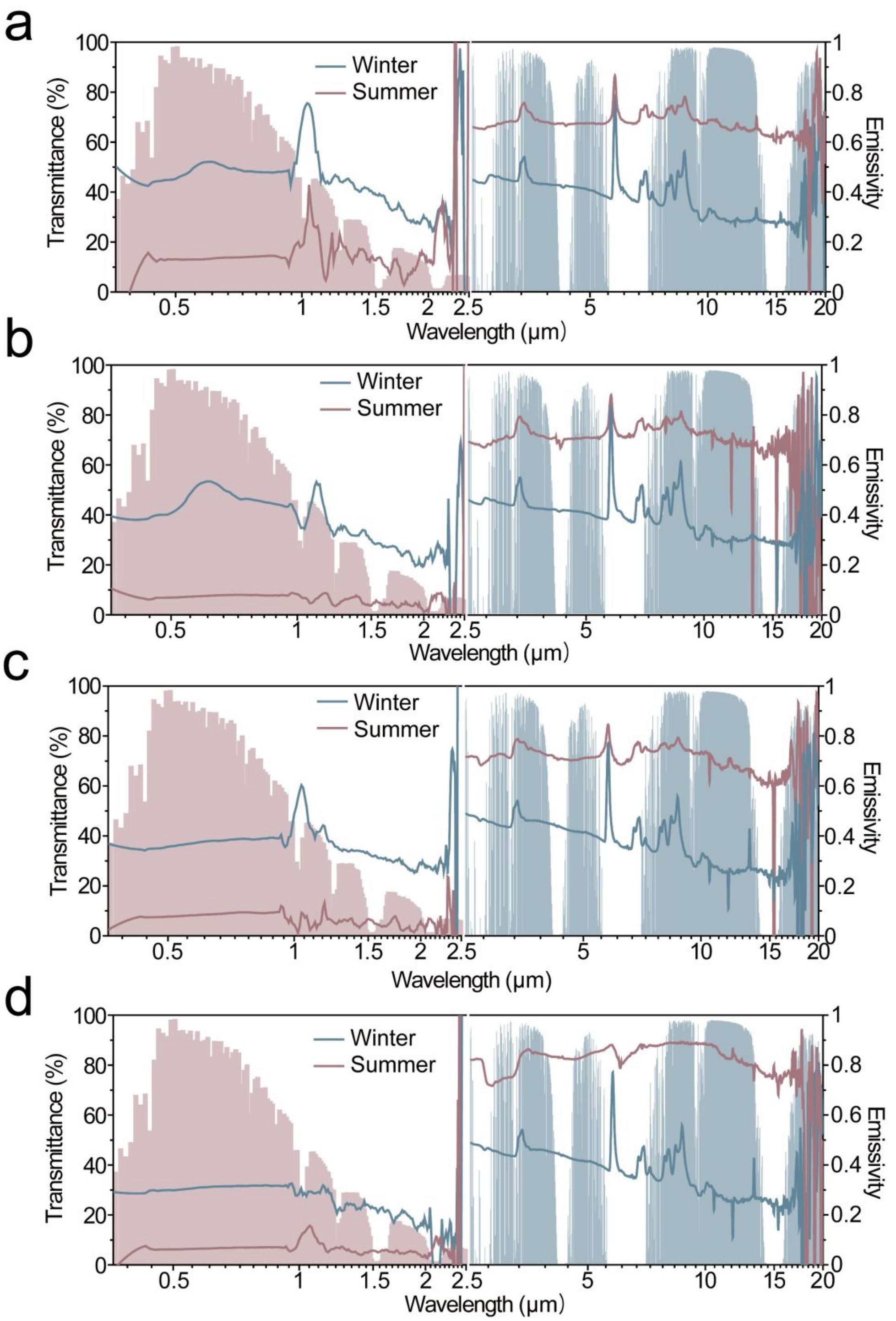

Figure S12. *UV-Vis-NIR and broadband IR emissivity spectra of the season-dependent smart window in the winter and summer application scenarios against a normalized AM1.5 global solar spectrum (Red shadow) and atmospheric transmittance window (Blue shadow), respectively (a: 25-second coating with 57 μm, b: 25-second coating with 90 μm, c: 25-second coating with 149 μm, d: 25-second coating with 209 μm).*

Analysis of optical and thermal radiation performance according to solar zenith angle of control group (stacked PMMA/ITO thin film on glass)

Figure S13a shows the optical properties of stacked PMMA/ITO thin film on glass according to the winter and summer application scenarios. Unlike the array sample, the control group does not show differences in light transmission characteristics depending on the application scenarios. Figure S13b shows the $\varepsilon_{\mathrm{Broadband}}$ characteristics of control group according to the winter and summer and the $\Delta\varepsilon_{\mathrm{Broadband}}$ is less than 0.1. Meanwhile, the IR image shows that there is no change in the color of the control group depending on the application scenarios (Figure S13c), indicating a strong reflectance of the control group independent of the winter and summer application scenarios. Figure S13d shows the UV-Vis-NIR and emissivity spectra of the control group across winter and summer scenarios. The control group demonstrates negligible solar modulation (less than 1%) and $\Delta\varepsilon_{\mathrm{Broadband}}$ (less than 0.1) between winter and summer scenarios, as indicated in the spectra.

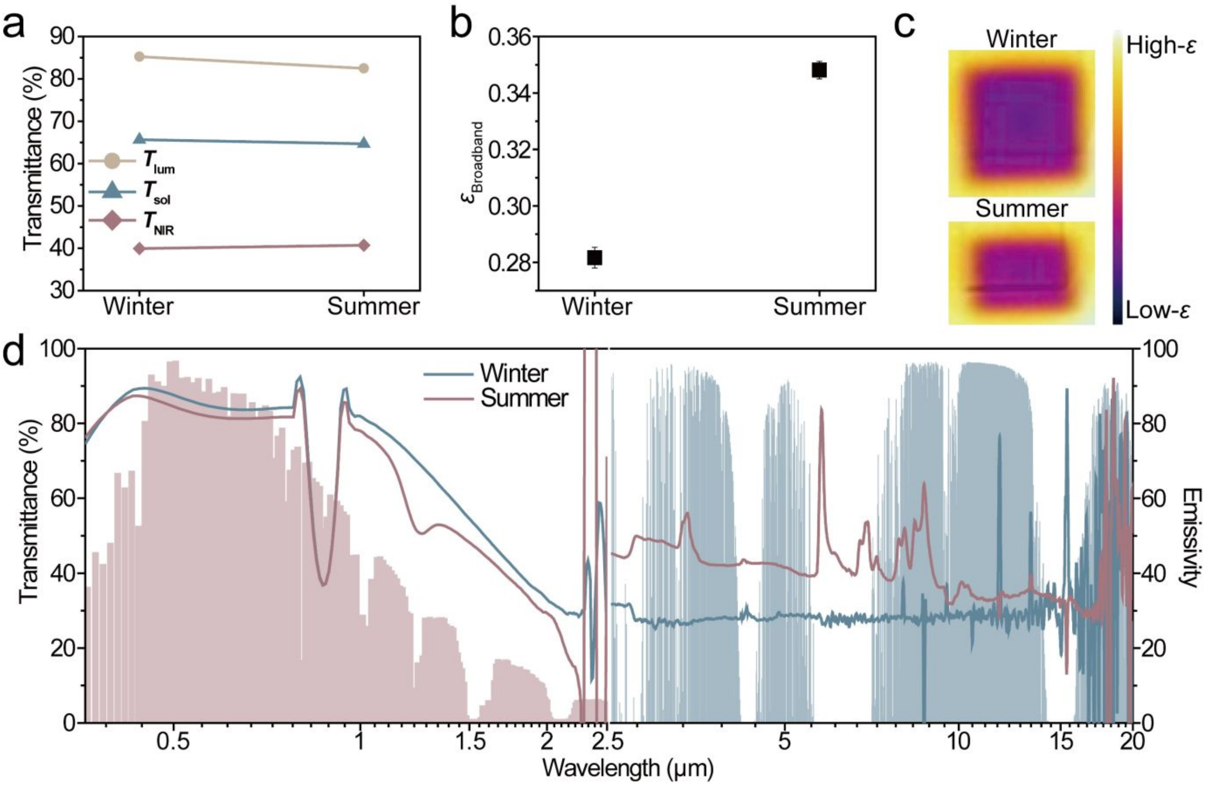

Figure S13. *(a) Optical performance of PMMA/ITO glass in the winter (θ: 90°, temperature: 30 °C) and summer (θ: 30°, temperature: 90 °C) application scenarios. (b) Emissivity according to the winter and summer application scenarios of PMMA/ITO glass. (c) IR images of PMMA/ITO glass for winter (Above) and summer (Below) application scenarios. (d) UV-Vis-NIR and emissivity spectra of the season-dependent smart window in the winter and summer application scenarios against a normalized AM1.5 global solar spectrum (Red shadow) and atmospheric transmittance window (Blue shadow), respectively.*

Various solar zenith angles and their impact on modulation abilities

Figure S14 demonstrates the modulation abilities of the periodic VO_2 array smart window under the various θ. The periodic VO_2 array smart window exhibits gradually decreasing transmittance as the θ decreases, at both LT and HT (Figures S14(a) and S14(b)). Simultaneously, $\varepsilon_{Broadband}$ progressively increases with decreasing θ under both LT and HT. These results confirm that the periodic VO_2 array structure effectively accommodates variations in θ, demonstrating its suitability for regions with distinct seasonal θ variations, particularly in northern latitude areas. Figure S15 presents the T_{sol} of the prototype as a function of θ (0° to 90°) under both LT and HT. At θ of 90° and LT, T_{sol} reaches a maximum of 38.1%. As the θ decreases—reflecting summer-like solar elevations—T_{sol} progressively declines, reaching a minimum of 4.5%.

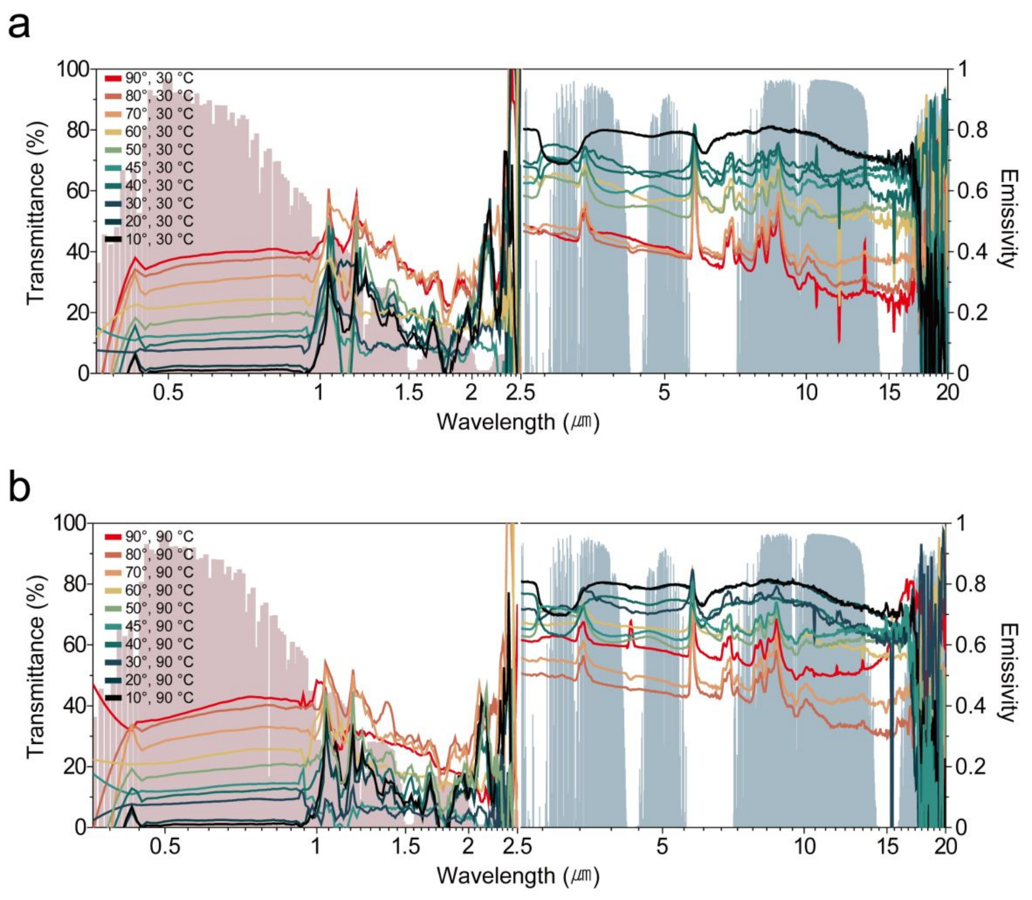

Figure S14. *UV-Vis-NIR and emissivity spectra of the dual-modulation smart window at various θ under (a) 30 °C and (b) 90 °C, overlaid with the normalized AM1.5 global solar spectrum (Red shading) and the atmospheric transmittance window (Blue shading), respectively.*

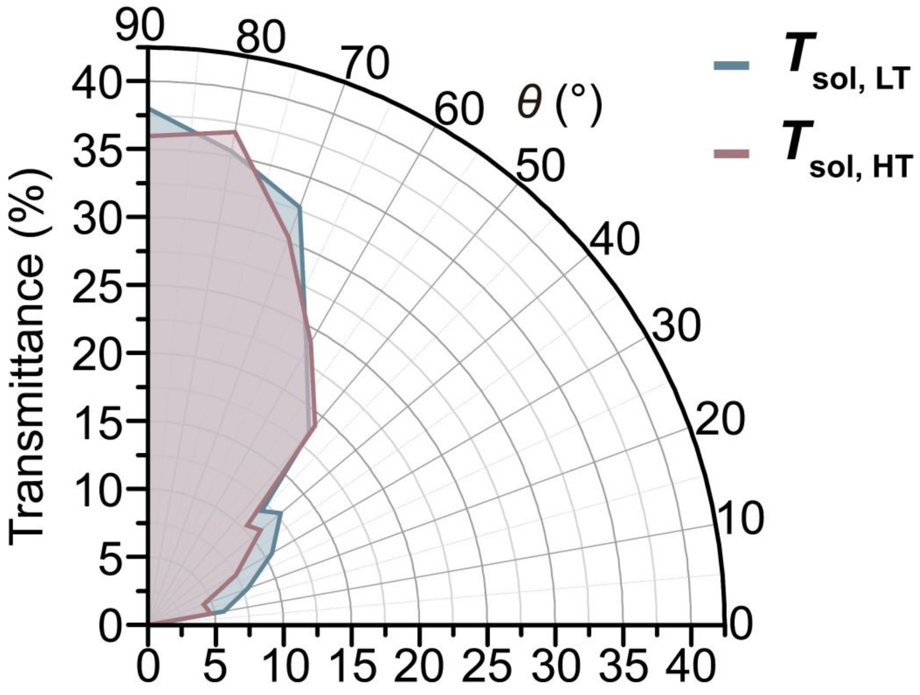

Figure S15. *Various angle-dependent solar modulation performance of the prototype at 30 °C and 90 °C*

Correlated color temperature (CCT) of continuous VO_2 film-based smart window

Figure S16 demonstrates the CCT of a conventional smart window with continuous VO_2 film. As shown in Figure S16, the continuous VO_2 film-based smart window has low CCTs (~4000 K) in both HT and LT. It indicates that the CCT of continuous VO_2 film is far from the daylight and it has a yellowish color.

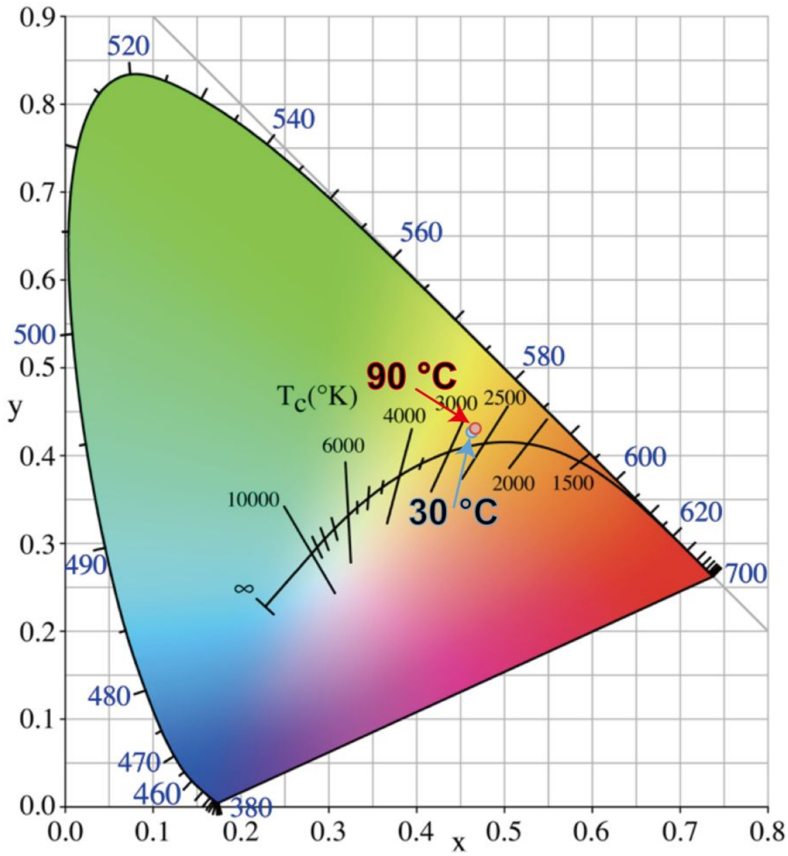

Figure S16. *Correlated color temperature (CCT) of conventional smart window based on continuous VO_2 film.*

Comparing energy saving performance of grating structure and the season-dependent dual-modulation smart window

The energy-saving performance of angle-dependent thermochromic grating structure reported previously[17] and the fabricated the periodic VO_2 array-based season-dependent dual-modulation smart window in actual-sized building are compared with the baseline of clear glass in cities with significant seasonal θ variability and ambient temperature difference—Seattle, London, and Seoul (Figure S17). In all three cities, dual-modulation smart window outperforms the grating structure with regards to annual energy-saving (Figure S17a). Figure S17b illustrates the monthly energy-savings of dual-modulation smart window and the grating structure in each city, with the clear glass as a baseline. Across all cities, dual-modulation smart window shows the best energy-saving performance, especially for the winter season (from January to March and from November to December). On the other hand, the grating structure shows a negative value in energy-saving for the winter season, due to its static, near-unity $\varepsilon_{Broadband}$. The observation of the performance differences between dual-modulation smart window and the grating structure highlights the importance of modulating both solar transmittance and outgoing thermal radiation. Figure S17c presents the winter and summer daylight illuminance of dual-modulation smart window and the grating structure in the three cities. Both the dual-modulation smart window and grating structure show effective daylight illuminance in the range of 100-2000 lux.

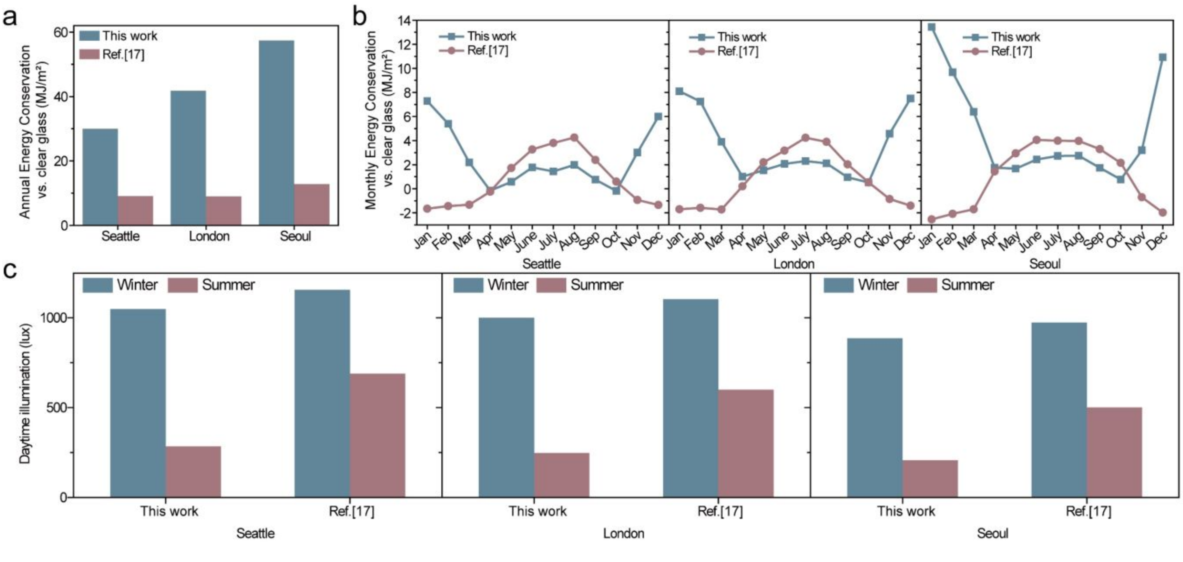

Figure S17. *Energy-saving performance and daylight illumination of the periodic VO_2 array-based*

season-dependent smart window and grating structure[17] *((a): Annual energy conservation, (b): Monthly energy conservation, and (c): Daylight illumination).*

Surface morphology and cross-sectional profiles of VO_2 arrays

Figure S18 shows optical microscope images, 3D surface topographies, and cross-sectional profiles of the arrays fabricated with different mesh opening sizes. The red arrow marks the region analyzed for cross-sectional height (Figure S18a). The optical images are consistent with the SEM observations of the VO_2 arrays. As shown in Figure S18b, the patterned VO_2 regions appear elevated relative to the flat areas. The arrays fabricated using 57 μm, 90 μm, 149 μm, and 209 μm meshes exhibit average heights (from valley to peak) of ~9.6 μm, 11.8 μm, 10.5 μm, and 10.5 μm, respectively.

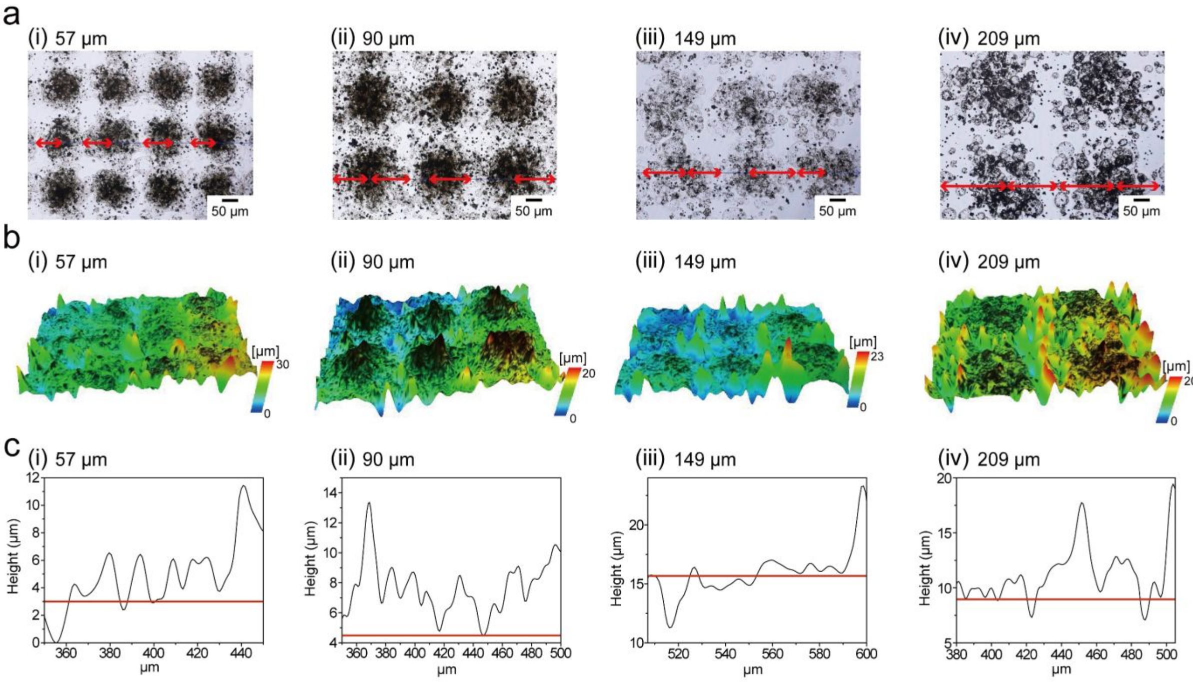

Figure S18. *Surface characterization of VO_2 arrays with different pitches: (a) Optical microscope images with the red arrow marking the analyzed cross-sectional path ((i) 57 μm, (ii) 90 μm, (iii) 149 μm, (iv) 209 μm). (b) Corresponding 3D surface topographies, where the color scale bar indicates surface height (red: higher regions, blue: lower regions). (c) Representative cross-sectional height profiles along the selected paths, with the red line denoting the baseline.*

Optical characteristics of clear glass and comparing energy consumption performance of clear glass and the season-dependent dual-modulation smart window

Figure S19a presents the UV–Vis–NIR transmittance and emissivity spectra of clear glass under winter and summer scenarios. The glass exhibits only minor transmittance modulation and almost no $\varepsilon_{\mathrm{Broadband}}$ change between winter and summer scenarios. Consistently, the IR images (Figure S19a, inset) show bright contrast in both winter and summer scenarios, further confirming persistently high emissivity. Figure S19b further illustrates that clear glass shows minimal seasonal variation in optical performance: ΔT_{sol}, ΔT_{NIR}, and ΔT_{lum} remain below ~5%, while $\Delta\varepsilon_{\mathrm{Broadband}}$ is nearly zero. Annual energy consumption simulation shows that clear glass exhibits substantially higher energy use across all regions compared to the proposed device (Figure S19c). Specifically, energy consumption with the device is ~220 MJ/m^2, ~200 MJ/m^2, and ~260 MJ/m^2 in Seattle, London, and Seoul, respectively, compared to ~260 MJ/m^2, ~250 MJ/m^2, and ~325 MJ/m^2 for clear glass. The maximum reduction reaches ~65 MJ/m^2. Figure S19d further shows consistently lower monthly energy consumption with the device, underscoring the critical role of responsiveness to seasonal θ and ambient temperature variations.

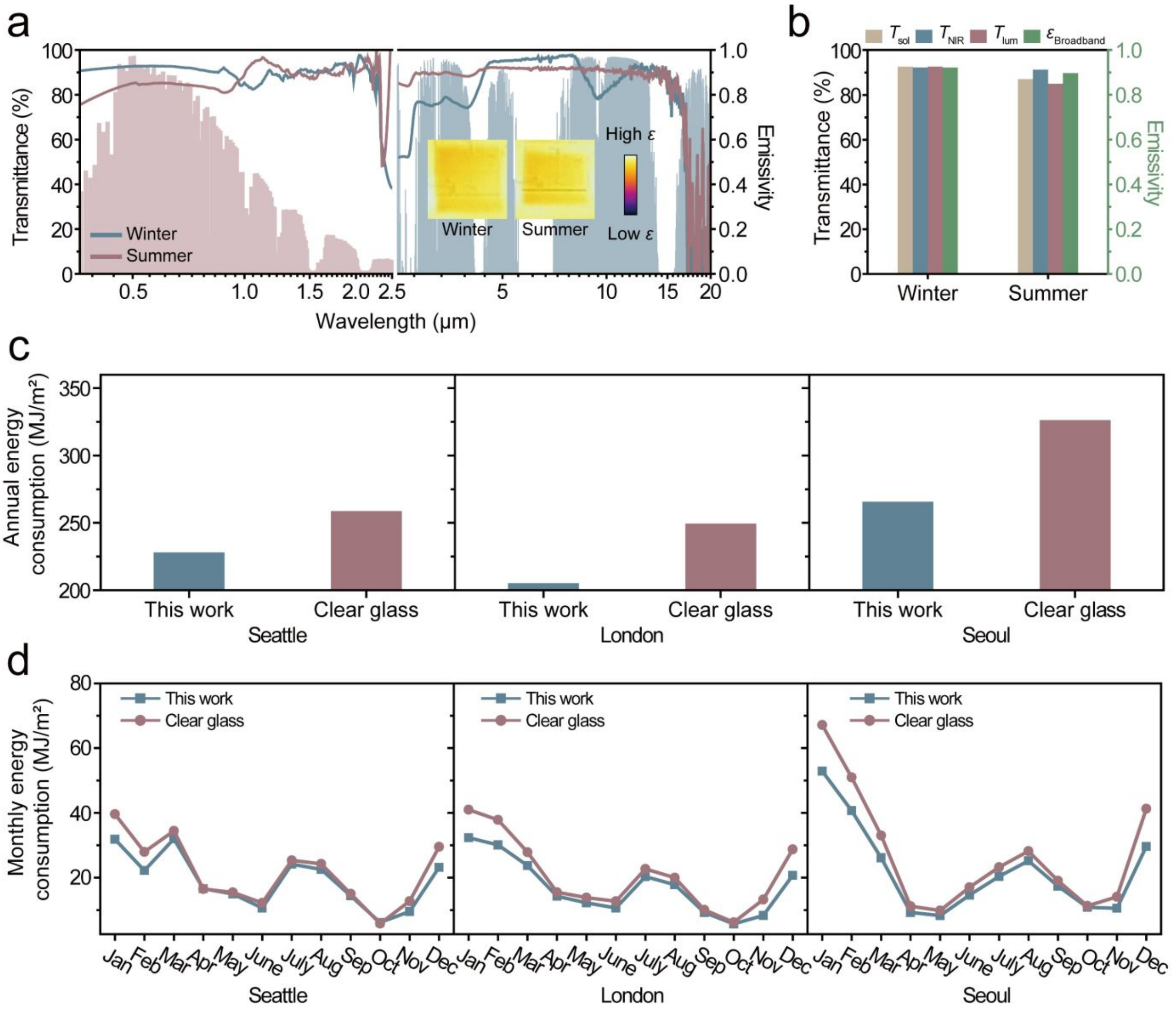

Figure S19. *(a) UV-Vis-NIR and emissivity spectra of the clear glass in the winter (θ: 90°) a nd summer (θ: 30°) application scenarios against a normalized AM1.5 global solar spectrum (Red shadow) and atmospheric transmittance window (Blue shadow), respectively. Inset: IR im ages of clear glass for winter (Left) and summer (Right) application scenarios. (b) Optical per formance of clear glass in the winter and summer application scenarios. (c) Annual energy consumption comparison of this work and clear glass. (d) Monthly energy consumption comparison of this work and clear glass.*

Table S1. Parameters of integrated mesh printing and spray-coating.

Acetone: PMMA %w/w	Coating temperature (°C)	VO_2 concentration (g)	Mesh opening size (μm)	Coating distance (cm)	Coating time (sec)
		0.25	57	7.5	15
		0.2625			
		0.275	90	10	20
0.791: 0.1 (12.6%)	60	0.3			
		0.3375	149	15	25
		0.375			
		0.45	209	20	30

Table S2. Building model specifications for energy-saving simulation.

Items	Specifications
Window fraction (window-to-wall ratio)	27.8% of above-grade gross walls
Window locations	Distribution among all four sides
Floor to ceiling height	2.7 m
Glazing sill height	0.35 m
Exterior walls	Wood siding + Fiberglass quilt + Plasterboard
Roof	Roof deck + Fiberglass quilt + Plasterboard
Window	The window type is described accordingly

Table S3. Optical properties of the periodic VO_2 island array smart window, angle-dependent thermochromic grating structure, planar control sample, low-E glass, and clear glass for energy-saving simulation (S: summer, W: winter application scenario).

Optical properties	This work	Angle-dependent thermochromic grating structure	Planar control sample	Commercial low-E glass	Clear glass
$\varepsilon_{\text{Broadband-W}}$	0.26	0.95	0.32	0.84	0.84
$\varepsilon_{\text{Broadband-S}}$	0.67		0.56		
$\Delta\varepsilon_{\text{Broadband}}$	0.41	0	0.24	0	0
$\varepsilon_{\text{Broadband-back}}$	0.84	0.84	0.1	0.49	0.84
$T_{\text{lum-W}}$ (%)	36.8	40.8	22.2	81.1	89.8
$R_{\text{lum-W-Front}}$ (%)	5.71	8.2	9.1	12.7	8.1
$R_{\text{lum-W-Back}}$ (%)	11.3	7.3	11.6	12.7	8.1
$T_{\text{lum-S}}$ (%)	8.0	19.5	22.2	81.1	89.8
$R_{\text{lum-S-Front}}$ (%)	5.3	8.2	9.1	12.7	8.1
$R_{\text{lum-S-Back}}$ (%)	10.7	7.3	11.6	12.7	8.1
$T_{\text{sol-W}}$ (%)	38.1	45.2	17.2	71.4	83.7
$R_{\text{sol-W-Front}}$ (%)	6.41	8.2	11.1	10.1	7.5
$R_{\text{sol-W-Back}}$ (%)	11.1	7.3	16.6	11.5	7.5
$T_{\text{sol-S}}$ (%)	7.3	21.9	12.0	71.4	83.7
$R_{\text{sol-S-Front}}$ (%)	7.83	8.2	7.7	10.1	7.5
$R_{\text{sol-S-Back}}$ (%)	13.7	7.3	16.5	11.5	7.5

Table S4. Optical properties and thermal radiation performance of this study and previously reported results on VO_2-based smart windows.

	This work	Grating VO_2[17]	Porous VO_2[18]	VO_2/Fabry-Perot resonator[11]	W-doped VO_2@SiO_2[19]
ΔT_{sol}	30.9%	25.8%	2.2%	9.3%	10.64%
T_{lum}	39.6%	43.3%	40.9%	27.8%	43.11%
$\Delta\varepsilon_{Broadband}$	0.41	0	0.4	0.4	0.23

<References>

[1] Wyszecki, G.; Stiles, W. S. *Color science: concepts and methods, quantitative data and formulae*; Wiley, Hoboken, NJ, **2000**; pp 1-959.

[2] G. Astm, *ASTM International* **2003**.

[3] Inoue, T.; Zoysa, M. D.; Asano, T.; Noda, S. Realization of dynamic thermal emission control. *Nat. Mater.* **2014**, 13, 928-931.

[4] Wu, S.-H.; Chen, M.; Barako, M. T.; Jankovic, V.; Hon, P. W.; Sweatlock, L. A.; Povinelli, M. L. Thermal homeostasis using microstructured phase-change materials. *Optica* **2017**, 4(11), 1390-1396.

[5] ANSI/ASHRAE/IES, "Energy standard for building except low-rise residential buildings" (ANSI/ASHRAE/IES, 2016); https://www.ashrae.org/file%20library/technical%20resources/standards%20and%20guidelines/standards%20addenda/90.1 2016/90_1_2016_m_ai_aj_au_az_bg_dn_20210324.pdf.

[6] Hunt, R. W. G.; Pointer, M. R. *Measuring colour*; Wiley, Hoboken, NJ, **2011**; pp 41-72.

[7] Ghosh, A.; Selvaraj, P.; Sundaram, S.; Mallick, T. K. The colour rendering index and correlated colour temperature of dye-sensitized solar cell for adaptive glazing application. *Sol. Energy* **2018**, 163, 537-544.

[8] C. TC, *CIE Pub.* **1988**, 75.

[9] McCamy, C. S. Correlated color temperature as an explicit function of chromaticity coordinates. *Color Res. Appl.* **1992**, 17(2), 142-144.

[10] C. I. de l'Eclairage, Tech. Rep., CIE https://cie.co.at/publications/Method of measuring and specifying colour rendering properties of light sources: Technical report: Cie 13.3-1995.

[11] Wang, S.; Jiang, T.; Meng, Y.; Yang, R.; Tan, G.; Long, Y. Scalable thermochromic smart windows with passive radiative cooling regulation. *Science* **2021**, 374(6574), 1501-1504.

[12] Kana Kana, J. B.; Vignaud, G.; Gibaud, A.; Maaza, M. Thermally driven sign switch of static dielectric constant of VO_2 thin film. *Opt. Mater.* **2016**, 54, 165-169.

[13] Tsuda, S.; Yamaguchi, S.; Kanamori, Y.; Yugami, H. Spectral and angular shaping of infrared radiation in a polymer resonator with molecular vibrational modes. *Opt.*

Express **2018**, 26, 6899-6915.

[14] König, T. A. F.; Ledin, P. A.; Kerszulis, J.; Mahmoud, M. A.; El-Sayed, M. A.; Reynolds, J. R.; Tsukruk, V. V. Electrically tunable plasmonic behavior of nanocube–polymer nanomaterials induced by a redox-active electrochromic polymer. *ACS Nano* **2014**, 8, 6182-6192.

[15] Rubin, M. Optical properties of soda lime silica glasses. *Sol. Energy Mater.* **1985**, 12, 275-288.

[16] Cleary, J. W.; Smith, E. M.; Leedy, K. D.; Grzybowski, G.; Guo, J. Optical and electrical properties of ultra-thin indium tin oxide nanofilms on silicon for infrared photonics. *Opt. Mater. Express* **2018**, 8, 1231-1245.

[17] Zhou, C.; Li, D.; Tan, Y.; Ke, Y.; Wang, S.; Zhou, Y.; Liu, G.; Wu, S.; Peng, J.; Li, A.; Li, S.; Chan, S. H.; Magdassi, S.; Long, Y. 3D printed smart windows for adaptive solar modulations. *Adv. Opt. Mater.* **2020**, 8(11), 2000013.

[18] Bhupathi, S.; Wang, S.; Wang, G.; Long, Y. Porous vanadium dioxide thin film-based Fabry− Perot cavity system for radiative cooling regulating thermochromic windows: experimental and simulation studies. *Nanophotonics* **2024**, 13(5), 711-723.

[19] Wang, Z.; Liang, J.; Lei, D.; Jiang, C.; Yang, Z.; Yang, G.; Zhang, D.; Zhang, L.; Zhang, C.; Bai, Y. Temperature-adaptive smart windows with passive transmittance and radiative cooling regulation. *Appl. Energy* **2024**, 369, 123619.
